# Cell Spheroids with Enhanced Aggressiveness to Mimic Human Liver Cancer *In Vitro* and *In Vivo*

**DOI:** 10.1038/s41598-017-10828-7

**Published:** 2017-09-05

**Authors:** Hong-Ryul Jung, Hyun Mi Kang, Jea-Woon Ryu, Dae-Soo Kim, Kyung Hee Noh, Eun-Su Kim, Ho-Joon Lee, Kyung-Sook Chung, Hyun-Soo Cho, Nam-Soon Kim, Dong-Soo Im, Jung Hwa Lim, Cho-Rok Jung

**Affiliations:** 1 Gene Therapy Research Unit, Korea Research Institute of Bioscience and Biotechnology(KRIBB), 125 Gwahak-ro, Daejeon, Republic of Korea; 2 Department of Functional Genomics, Korea university of Science and Technology (UST), 217 Gajeong-ro, Daejeon, Republic of Korea; 3 Genome Research Center, Korea Research Institute of Bioscience and Biotechnology(KRIBB), 125 Gwahak-ro, Daejeon, Republic of Korea

## Abstract

We fabricated a spheroid-forming unit (SFU) for efficient and economic production of cell spheroids. We optimized the protocol for generating large and homogenous liver cancer cell spheroids using Huh7 hepatocellular carcinoma (HCC) cells. The large Huh7 spheroids showed apoptotic and proliferative signals in the centre and at the surface, respectively. In particular, hypoxia-induced factor-1 alpha (HIF-1α) and ERK signal activation were detected in the cell spheroids. To diminish core necrosis and increase the oncogenic character, we co-cultured spheroids with 2% human umbilical vein endothelial cells (HUVECs). HUVECs promoted proliferation and gene expression of HCC-related genes and cancer stem cell markers in the Huh7 spheroidsby activating cytokine signalling, mimicking gene expression in liver cancer. HUVECs induced angiogenesis and vessel maturation in Huh7 spheroids *in vivo* by activating epithelial–mesenchymal transition and angiogenic pathways. The large Huh7 cell spheroids containing HUVECs survived at higher concentrations of anti-cancer drugs (doxorubicin and sorafenib) than did monolayer cells. Our large cell spheroid provides a useful *in vitro* HCC model to enable intuitive observation for anti-cancer drug testing.

## Introduction

Currently, monolayer cell cultures are the most commonly used models for *in vitro* cancer drug testing. Traditional two-dimensional (2D) models have significantly contributed to cancer research. However, they cannot mimic *in vivo* three-dimensional (3D) tumour growth, with specific architecture and various signals governing cellular processes. Multicellular spheroids are one of the most widely used models for 3D cell culture, and various culture methods and tools, such as devices that provide physical forces like gravity or rotation, have been developed^1, 2^. However, these techniques require expensive equipment, and generating homogenous and large spheroids remains difficult^3, 4^. Recently, researchers have developed enhanced techniques for drug screening supporting 3D cell culture on a high-throughput scale^5^ and with uniform size^6^. Although the reliability of 3D versus 2D culture has been well established, efficient and economic tools for fabricating large, homogenous 3D cell spheroids are still needed.

Hepatocellular carcinoma (HCC) occurs worldwide, with the highest incidence in Asian countries^7^. HCC is associated with poor prognosis because early diagnosis and treatment are not fully developed^8, 9^. Furthermore, the mechanisms underlying tumourigenicity in HCC remain unknown. Current investigations on HCC focus on the development of suitable model systems that can be used to increase our understanding of the disease mechanisms and to develop therapeutic tools^10^. Huh7 is a well-established carcinoma cell line derived from differentiated hepatocytes^11^. Here, we developed and optimized a tool, which we termed ‘spheroid-forming unit’ (SFU), for generating large-size multicellular cell spheroids, using Huh7 cells and human umbilical vein endothelial cells (HUVECs). More specifically, we aimed to produce a large-size cell spheroid mimicking the human liver cancer and provide *in vitro* HCC model for anti-cancer drug test.

## Results

### Generation of a large-size spheroid reflecting the tumour cellular environment

**Figure 1:**
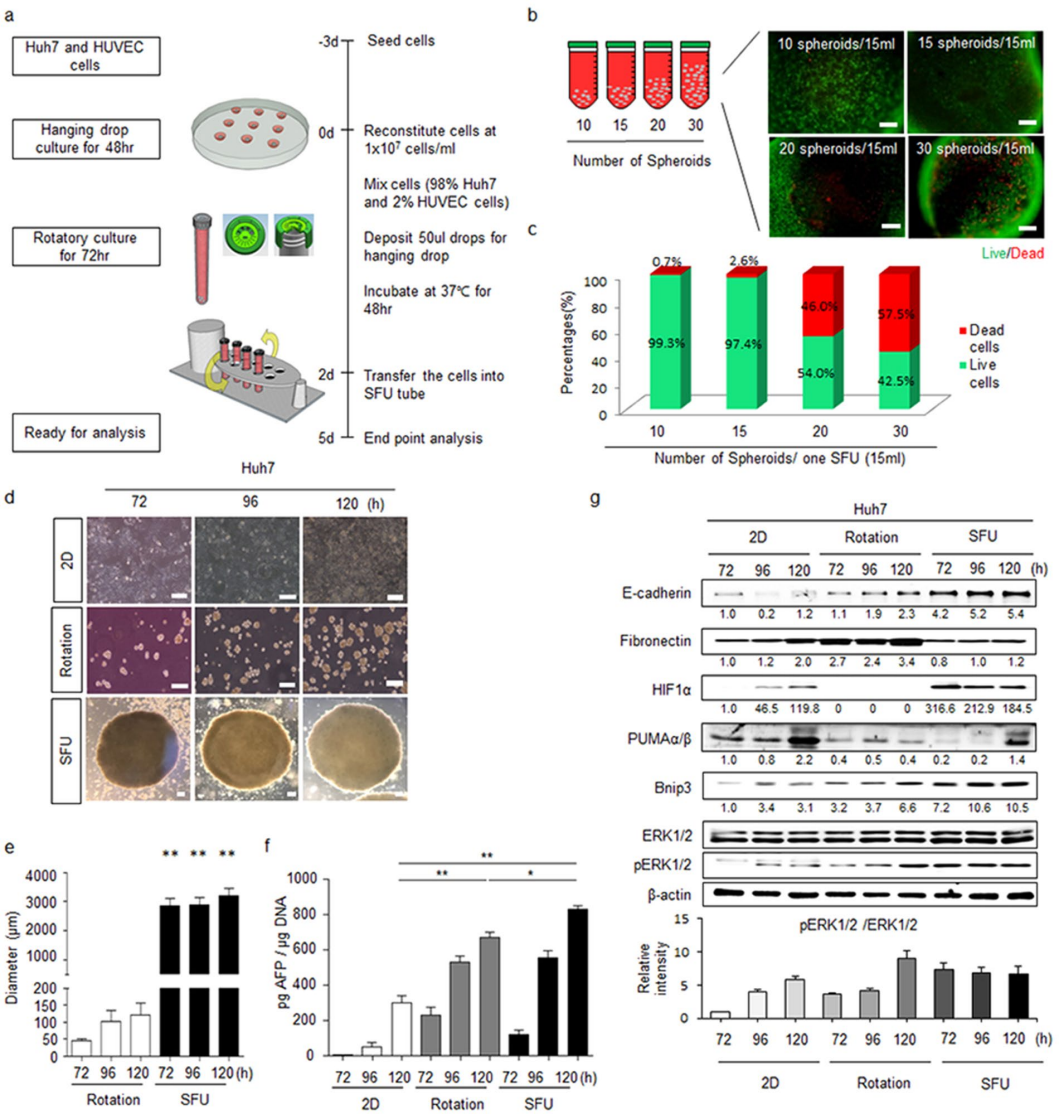
Biological characteristics of the SFU-based Huh7 spheroid. (**a**) Experimental procedure for cell spheroid production. (**b**) Live/dead stained image of spheroids incubated in 10, 15, 20, and 30 drops per 15 ml of medium. Green and red colours represent living and dead cells, respectively. Scale bars, 200 μm. (**c**) Percentage of live and dead cells in the spheroids under the indicated conditions. (**d**) Representative DIC images of time-course analysis of cells generated by 2D plate culture, rotary culture, and the SFU. Scale bars, 200 μm. (**e**) Diameters of cell spheroids generated by rotary culture and the SFU for 72, 96, and 120 h. (**f**) ELISA of AFP secretion in culture supernatant of cell spheroids generated by rotary culture and the SFU for 72, 96, and 120 h. (**g**) Time-course of the expression of ECM, HIF-1α, apoptosis and proliferation signalling proteins in monolayers (2D) and spheroids generated by rotary culture and the SFU as assessed by western blotting. *P < 0.05; **P < 0.01.

To efficiently and economically establish size-controlled cell spheroids, we designed a protocol combining both the hanging-drop and rotation approaches to fabricate an SFU consisting of a tube and filter cap. In brief, we deposited 50-μl droplets containing 5 × 10^5^ Huh7 cells onto the lower side of a Petridish lid after which the lid was flipped onto the dish, which was filled with PBS to prevent evaporation. After a 48-h incubation, we transferred cell aggregates to SFUs filled with 15 ml of medium for an additional 72-h rotary culture (Fig. 1a). In addition, we also examined whether large spheroids could be generated by other methods such as stationary culture after hanging drop and Ultra-Low Attachment Surface plate (Supplementary Fig. S1a). Compared to the spheroid of SFU, dead cells were markedly higher in those of stationary culture and ultra-low attachment plate (Supplementary Fig. S1a). Some of the spheroids produced by stationary culture were shrunken, punctured, or had scattered cells (Supplementary Fig. S1b) at 120 h of culture. Moreover, using an ultra-low attachment plate with the same initial number of cells as that used in the SFU protocol, the cells did not aggregate and were easily dispersed, in contrast the spheroid cultured with lower cell numbers (2 × 10^4^ cells according to the manufacturer’s instructions) showed healthy and well-formed cell spheroid (Supplementary Fig. S1c). Based on these findings, we further optimized the SFU protocol.

To test how many spheroids can be cultured in a SFU, we transferred 10 to 30 cell aggregates and cultured as described above. Live/dead staining indicated 10–15 spheroids per SFU to be optimal to obtain healthy spheroids (Fig. 1b,c). Additionally, we tested the usability of the SFU for various other cell lines, including Hep3B, HepG2, CK-K1, and SK-Hep1. We successfully obtained spheroids for all lines by combining the hanging-drop method and SFU (Supplementary Fig. S2a). Huh7 cells were found to form spheroids more easily than other cell types. Therefore, and because of their relevance as a cancer model, we used Huh7 spheroids for further experiments. After 120 h of culture, Huh7 spheroids had a diameter of ~3 mm, which was larger than that of spheroids produced by rotation alone with the same amount of starting material (Fig. 1d,e). We compared the biological and oncogenic characteristics of Huh7 spheroids produced with the aid of the SFUs with those of spheroids generated by 2D stationary or rotary culture alone. As alpha-fetoprotein (AFP) is commonly elevated in HCC, we examined the amount of AFP secreted by spheroids. SFU- and rotation only (3D)-based spheroids showed higher AFP secretion than monolayers (Fig. 1f). Additionally, we analysed changes in the extracellular matrix (ECM) components fibronectin and E-cadherin. Fibronectin is a major component of the ECM and E-cadherin plays an important role in cell adhesion by forming cellular junctions. Compared to 2D culture, fibronectin was significantly higher in rotation-based, but not in SFU-based spheroids, while E-cadherin was significantly higher in the SFU-based spheroids (Fig. 1g). These data showed that rotary culture as well as the SFU promotes ECM expression and AFP secretion in the Huh7 spheroids. Because the SFU-based spheroids were larger than the rotary culture-based spheroids, we expected the nutrient and oxygen gradients, and consequently, protein expression, to be affected. Hypoxia-inducible factor-1α (HIF-1α) and PUMAα/β, a pro-apoptosis protein, were detected in cells at the centre of the spheroid, while most cells at the periphery of the SFU-based spheroid were alive after 120 h (Supplementary Fig. S2b). In addition, we analysed ERK signalling activation to confirm the proliferative status of the SFU-based spheroid; pERK was higher at the early time points of 72 and 96 h in SFU-based spheroids than in monolayers or rotary culture-based spheroids (Fig. 1g). These results suggested that cell proliferation occurred mostly near the surface. On the other hand, apoptosis occurred in the centre of the spheroids. Therefore, we further optimized the protocol to generate healthy large Huh7 cell spheroids.

### HUVECs at 2% suffice to promote oncogenic properties of the Huh7 spheroids

The observation of a necrotic core in the SFU-based Huh7 spheroids inspired us to co-culture the spheroids with endothelial cells (HUVECs or liver-specific endothelial T0056 cells) or fibroblasts (MRC-5 cells) with the aim to support Huh7 survival and maturation. Spheroids co-cultured with HUVECs, T0056, or MRC-5 cells commonly showed higher pERK expression. However, Huh7 spheroids co-cultured with HUVECs showed the lowest expression of the apoptosis-related proteins PUMAα/β and Bnip3 than spheroids of the other culture methods (Supplementary Fig. S3a). These results indicated that HUVECs enhance proliferation and decrease apoptotic signals in the Huh7 spheroids. Moreover, HUVECs enhanced the mRNA expression as well as secretion of VEGF and AFP by the Huh7 spheroids (Supplementary Fig. S3b–d). Reverse transcription quantitative (RT-q)PCR analysis showed that HUVECs promoted gene expression related to cell proliferation, including cyclins (Supplementary Fig. S3e), and HCC-associated oncogenes such as *IGF2, cMET, RHOA, TCF4, TNFSF10*, and *TGFA* (Supplementary Fig. S3f), which was verified by conventional RT-PCR (Supplementary Fig. S3g). Thus, HUVECs induced oncogenic properties more effectively than the other cell lines and were therefore used in further experiments.

**Figure 2:**
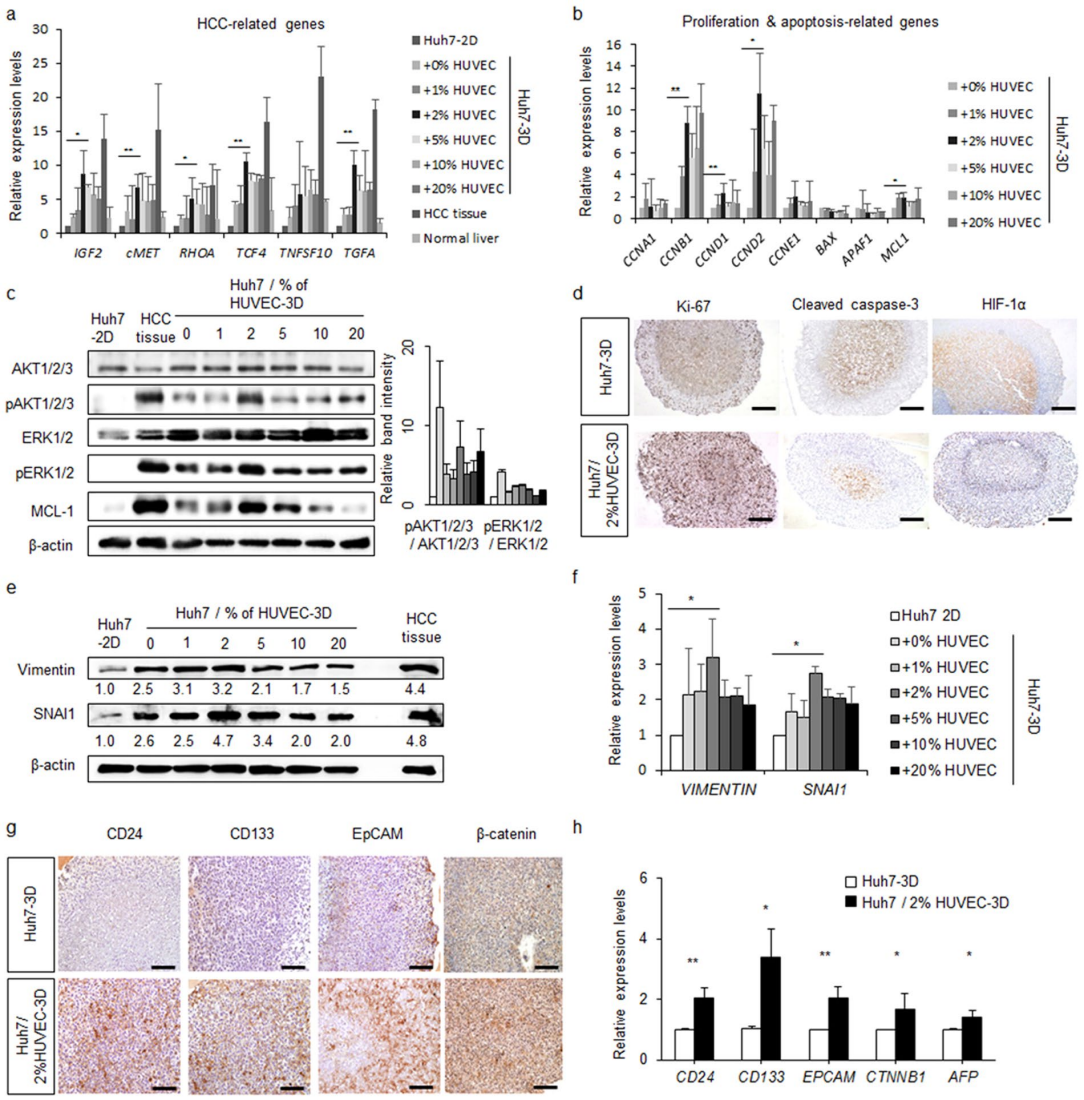
HUVECs enhance the survival of Huh7 spheroid by HCC-related gene and cancer stem cell marker expression. (**a**) RT-qPCR analysis of tumour-related genes in the spheroids co-cultured with the indicated percentages of HUVECs at 120 h in comparison to cancer/normal liver tissues and monolayers (2D). (**b**,**c**) Proliferation (cyclins and AKT/ERK pathway) and apoptosis signalling (*BAX, APAF1*, and *MCL1*)-related mRNA (**b**) and proteins (**c**) as detected by RT-qPCR and western blotting, respectively. (**d**) IHC of Huh7-3D or Huh7/2% HUVEC-3D for proliferation (Ki-67) and apoptosis (cleaved caspase-3) markers. Scale bars, 200 μm. (**e**,**f**) SNAI1 and vimentin, and EMT-related proteins (**e**) and mRNA (**f**) were detected by western blotting and RT-qPCR, respectively. (**g**,**h**) Cancer stem cell markers (CD24, CD133, EpCAM, and β-catenin) and AFP were analysed by IHC of Huh7-3D or Huh7/2% HUVEC-3D (**g**) and RT-qPCR (**h**). Scale bars, 200 μm. *P < 0.05; **P < 0.01.

To determine the appropriate proportion of HUVECs in Huh7 spheroid culture, we cultured spheroids with 0%, 1%, 2%, 5%, 10%, and 20% of HUVECs in total cells for 120 h. As a control, we attempted to generate spheroids using HUVECs alone; however, the cells did not aggregate. Moreover, most cells died after rotatory culture for 120 h, not allowing proteome analysis (Supplementary Fig. S4a). Additionally, we checked GFP expression by flow cytometry in Huh7 spheroids co-cultured with 0%, 1%, 2%, 5%, 10%, and 20% of GFP-tagged HUVECs at 120 h to determine the actual proportion of HUVECs in 3D spheroids (Supplementary Fig. S4b). RT-qPCR data showed that 2% HUVECs increased HCC-related oncogenes. Particularly, the expression of *IGF2*, *cMET*, *TCF4*, and *TGFA* was significantly increased in the presence of 2% HUVECs, as was that of cyclins B1 and D2 (Fig. 2a,b). Expression of all of the tested genes was confirmed by conventional RT-PCR (Supplementary Fig. S5). Moreover, western blot results showed that the expression of proliferation-related and anti-apoptotic proteins was enhanced in spheroids co-cultured with 2% HUVECs (Fig. 2c). pERK and pAKT were significantly up-regulated in 3D culture as compared to 2D cells, and 2% HUVECs had an enhancing effect. To identify critical signalling pathway to cell proliferation in Huh7 spheroids, we tested the effects of MAPK and PI3K inhibitors on proliferation; both these pathways played a role in the survival of spheroids. In particular, the PI3K-Akt signalling pathway was relatively more significant to the viability of the spheroids (Supplementary Fig. S6). The proliferation marker Ki-67 was detected on the surface of Huh7 spheroids cultured without HUVECs, but when the spheroids were co-cultured with HUVECs, it was detected throughout the spheroid (Fig. 2d). The opposite was noted for the apoptotic and hypoxia markers, cleaved caspase-3 and HIF-1α; in spheroids generated with Huh7 alone, the marker was expressed in a large central area of the spheroid, while in co-culture, expression was more limited to the centre (Fig. 2d). We tested then effect of HUVECs in various liver cancer cell lines in addition to Huh 7 cell line. In result, it also increased HCC-associated, pro-proliferation and anti-apoptotic gene expression in a different cell spheroid respectively. (Supplementary Fig. S7). Epithelial–mesenchymal transition (EMT) is a cellular developmental program characterized by loss of cell adhesion and increased cell mobility, and it is essential for numerous processes including metastasis. Expression of SNAI1 and vimentin, factors relevant to EMT, was significantly higher in spheroids co-cultured with 2% of HUVECs than in spheroids of Huh7 alone (Fig. 2e,f). mRNA and protein levels of the tumourigenic cancer stem cell markers CD24, CD133, EpCAM, and β-catenin were higher in the spheroids cultured with HUVECs (Fig. 2g,h). Thus, 2% of HUVECs in Huh7 spheroids enhanced cell proliferation, EMT, and cancer stem cell marker expression. We suggest that these phenomena provided the Huh7 spheroid with tumorigenic aggressiveness *in vitro*.

### Huh7 spheroids generated by 3D co-culture with HUVECs mimic gene expression in human liver-cancer tissues

**Figure 3:**
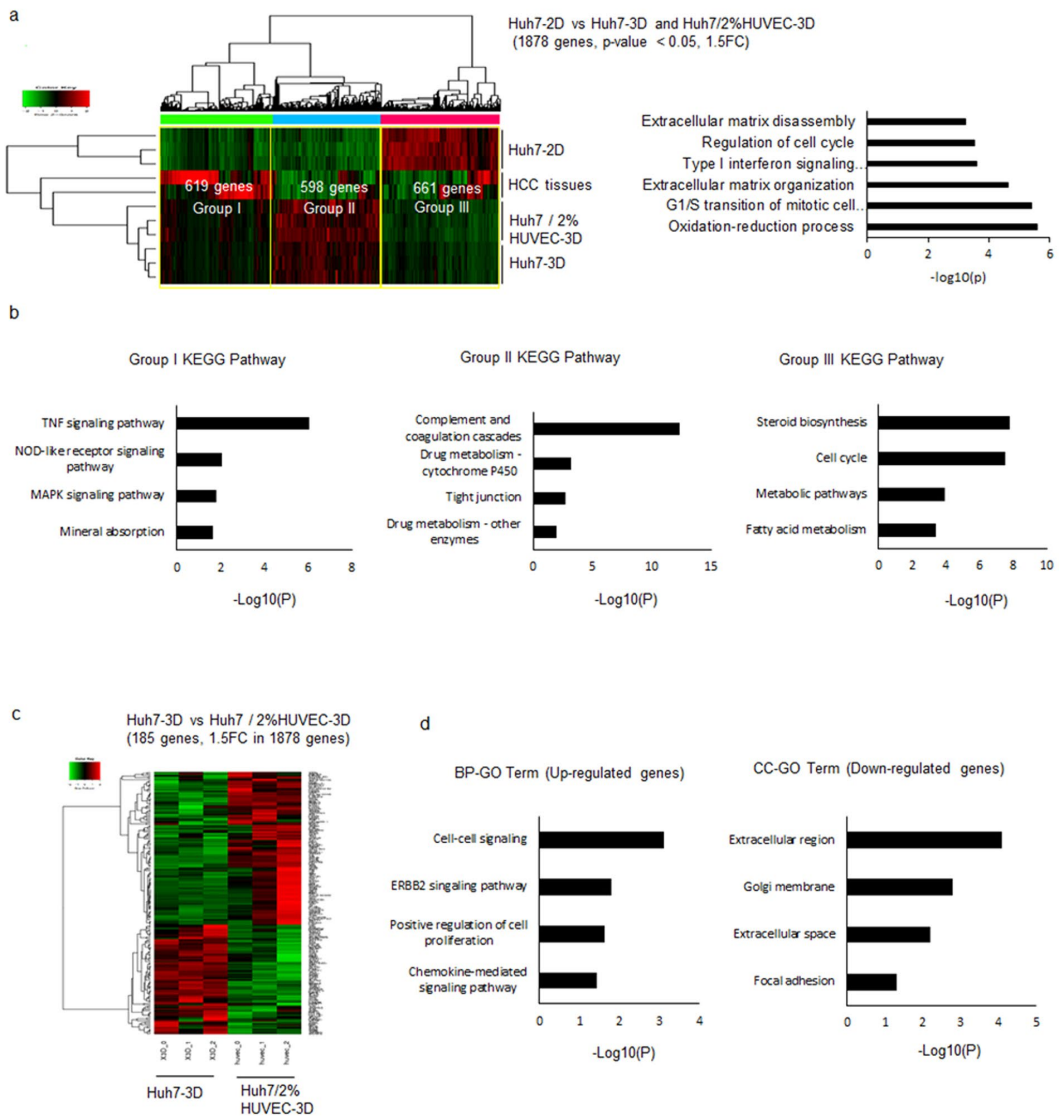
Huh7 spheroid containing HUVECs show similar gene expression profile to human liver cancer. (**a**) Heat-map showing comparative RNA expression levels and gene ontology analysis in 2D plate culture, rotary culture, SFU, and liver cancer tissues. Colours indicate the degree of up-regulation (red) and down-regulation (green) of RNA levels. (**b**) Classification of up-regulated (groups I and II) and down-regulated (Group III) genes in Huh7/2% HUVEC-3D as compared to 2D and their KEGG pathways, and heat-map of RNA expression related to a representative pathway in each group; drug metabolism in group I, MAPK signalling pathway in group II, and a metabolic pathway in group III. (**c**) Heat map showing comparative RNA expression in Huh7/2% HUVEC-3D as compared to Huh7-3D. (**d**) Classification of up-regulated and down-regulated genes in the GO terms of Huh7/2% HUVEC-3D compared to Huh7-3D.

We performed RNA sequencing (RNA-seq) analysis to compare the gene expression profiles of Huh7 spheroids and human liver cancer, and to identify the effects of 2% HUVECs. We identified 1,878 genes with a corrected *P-*value of 0.05 and at least a 1.5-fold change in expression in Huh7-3D and Huh7/2% HUVECs-3D as compared to Huh7-2D. Gene clustering revealed that Huh7-3D as well as Huh7/2% HUVECs-3D were more similar to human cancer tissue than Huh7-2D (Fig. 3a). High-level pattern analysis using gene ontology tools highlighted the differential regulation of 3 key pathways; cell cycle, extracellular matrix organization, and oxidation-reduction processes (Fig. 3a). We divided the differentially expressed genes into 3 groups according to their expression pattern. Group I genes were more strongly expressed in both Huh7-3D and Huh7/2% HUVECs-3D and human liver cancer tissues than in 2D-cultured cells; they were related to drug metabolism, and included genes encoding cytochrome P450s (Fig. 3b, Supplementary Fig. S8). Group II genes, which were highly expressed in both types of Huh7 spheroids as compared to human liver cancer tissues, were related to cytokine-related signalling, including TNF and MAPK signalling (Fig. 3b, Supplementary Fig. S8). Group III genes were expressed significantly lower in spheroids as well as liver cancer tissues than in Huh7-2D and were mostly related to metabolic pathways (Fig. 3b, Supplementary Fig. S8). 3D cell culture induced gene expression in Huh7 spheroids similar to that observed in human liver cancer. To identify the effect of HUVECs, we analysed fold changes of gene expression between Huh7-3D and Huh7/2% HUVECs-3D. Among the 1,878 genes, 187 were differentially expressed with >1.5-fold changes between Huh7-3D and Huh7/2% HUVECs-3D (Fig. 3c, Supplementary Tables S2 and S3). Genes related to cell-cell signalling, proliferation, and chemokine-mediated signalling pathways were up-regulated in the presence of HUVECs (Fig. 3d, Supplementary Table S2). On the other hand, extracellular region- and matrix-related genes were down-regulated in the presence of HUVECs (Fig. 3d, Supplementary Table S3). In short, 3D cell culture with HUVECs generated HCC-mimicking gene expression profiles in Huh7 spheroids, with enhanced cell-cell signalling and reduced extracellular compartment-related expression.

### HUVECs play a role in survival and invasion of Huh7 spheroids via cytokine-mediated signalling *in vitro*

**Figure 4:**
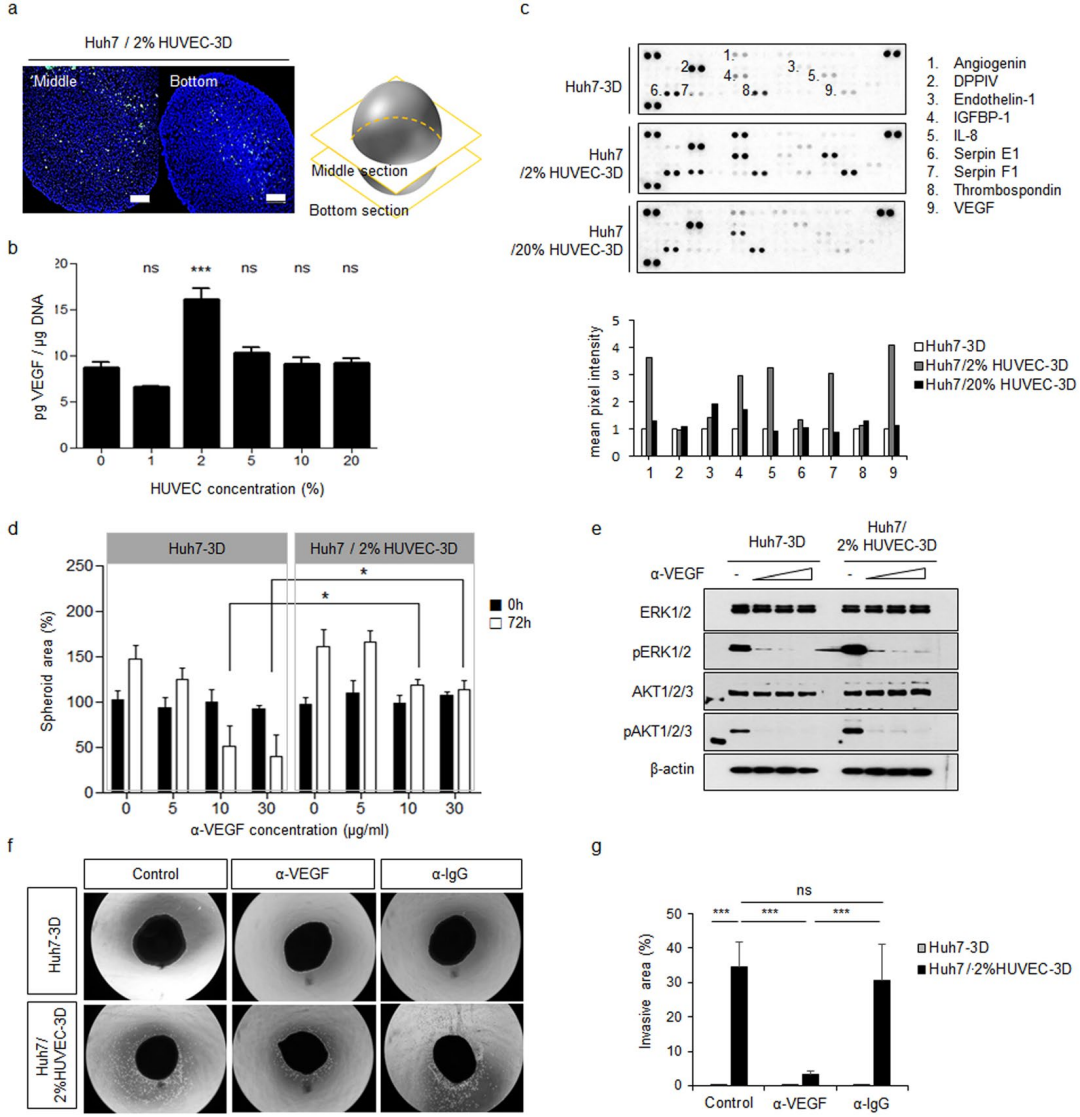
HUVECs promote proliferation and invasion by activation of MAPK pathway via cytokine-mediated signalling *in vitro*. (**a**) Distribution of 2% of HUVECs in the spheroid was analysed using ICC images of middle and bottom sections of cell spheroids co-cultured with 2% of GFP-tagged HUVECs. Scale bars, 200 μm. (**b**) ELISA of VEGF secretion in culture supernatant in spheroids co-cultured with the indicated concentrations of HUVECs as compared with Huh7 spheroids cultured alone, at 120 h. (**c**) Human cytokine array analysis of Huh7 spheroids cultured alone, with 2% HUVECs, and with 20% HUVECs. (**d**) Huh7-3D or Huh7/2% HUVEC-3D were transferred as single spheroids into each well of 96-well plate and treated with serum-free DMEM containing VEGF-neutralizing antibody (5, 10, 30 µg/ml) for 72 h. Spheroid areas were quantitated using ImageJ software before adding VEGF antibody (0 h) and after 72 h. Error bars display the standard deviation of five independent measurements. (**e**) At 72 h after VEGF antibody treatment, spheroids of each experimental group were harvested and analysed by western blotting with the indicated antibodies. (**f**) Huh7-3D or Huh7/2% HUVEC-3D were embedded into Matrigel in a 24-well plate and treated with serum-free DMEM containing 30 µg/ml VEGF-neutralizing antibody for 5 days. Mouse IgG isotype control antibody (30 µg/ml) was used as negative control. Representative images of each group are shown. (**g**) The sprouting area per experimental group was quantitated with ImageJ. Error bars display the standard deviation of six independent measurements. *P < 0.05; ***P < 0.001.

We expected that HUVECs would provide a vascular environment to the spheroids. However, 2% HUVECs did not induce the formation of a perivascular structure but were rather spread throughout the spheroid structures (Fig. 4a). As VEGF is the best-known cytokine regulating signal pathways in endothelial and cancer cells, we tested the secretion of VEGF using various concentrations of HUVECs; 2% HUVECs effectively increased VEGF secretion (Fig. 4b). To test the effect of cytokines derived from the HUVECs on Huh7 cell proliferation, Huh7 cells were seeded with/without HUVECs in a double-layered permeable culture dish. After 5 days, Huh7 cells cultured with HUVECs were dramatically more abundant than Huh7 cells cultured alone (Supplementary Fig. S9a,b). Together, these findings indicated that 2% HUVECs did not suffice to induce a vascular architecture but stimulated extracellular factor-mediated proliferation.

To identify cytokine(s) other than VEGF involved in the enhancement of cell proliferation in the spheroids, we performed a cytokine array analysis. Various cytokines related to angiogenesis, such as angiogenin, IGFBP-1, serpin F1, and IL8, in addition to VEGF, were increased under co-culture with 2% HUVECs (Fig. 4c). Moreover, Huh7 cells co-cultured with 20% HUVECs secreted less factors than spheroids cultured with 2% HUVECs, did not form intact spheroids, and showed reduced cell growth during starvation for preparing cytokine array analysis (data not shown). Next, we tested the effects of cytokines on the growth and invasion of Huh7 spheroids. Before performing these assays, we confirmed that the presence of HUVECs promoted proliferation and invasion signalling in Huh7 spheroids at the protein level (Supplementary Fig. S9c). One Huh7 spheroid was added to each well of a 96-well plate containing specific antibodies, and the areas of the spheroids were analysed after 72 h and ATP was measured to ensure the correlation between reduction of areas and survival of spheroids (Fig. 4d, and Supplementary Fig. S10a,b). Blocking of VEGF with specific antibodies reduced spheroid survival (Fig. 4d). Huh7/2% HUVECs-3D were more resistant to such antibody treatment than Huh7-3D (Fig. 4d). Anti-VEGF antibody, which blocks signalling downstream of VEGFR, inhibited activation of ERK and AKT signals (Fig. 4e). Similar results were obtained for spheroids in which angiogenin was blocked using specific antibodies (Supplementary Fig. 10c–e). We next carried out an invasion assay using Huh7-3D and Huh7/2% HUVECs-3D. From the initial stage, Huh7/2% HUVECs-3D was more invasive than Huh7-3D; thus, the effect of inhibition of invasion was clearer for Huh7/2% HUVECs-3D than for Huh7-3D (Fig. 4f,g). We concluded that 2% HUVECs supported the survival and invasiveness of the Huh7 spheroids by activating the MAPK pathway *via* stimulating extracellular cytokine signalling *in vitro*.

### HUVECs promote vascularization and aggressiveness of Huh7 spheroids *in vivo*

**Figure 5:**
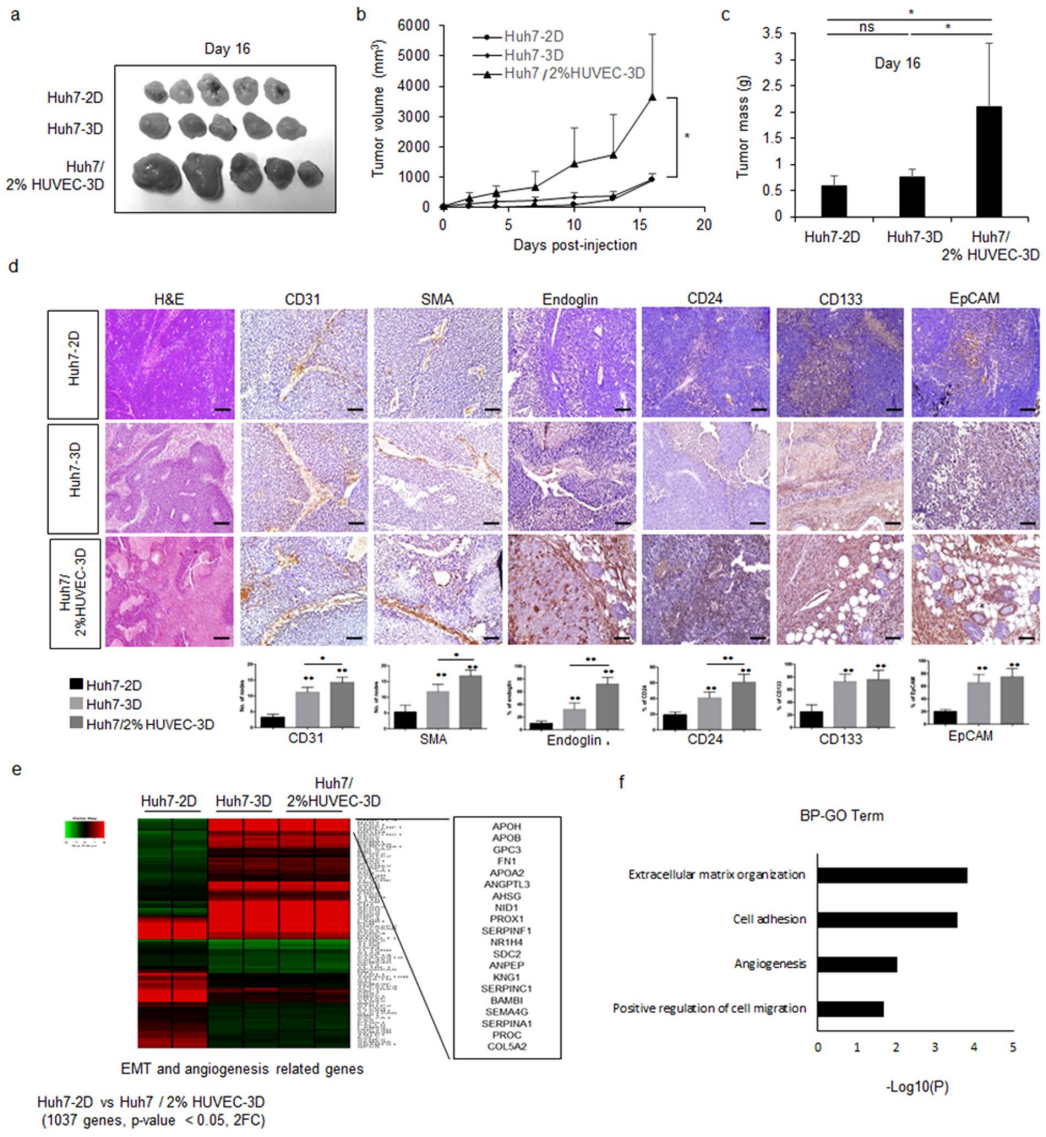
HUVECs enhance tumour growth and vascularization by up-regulation of EMT and angiogenesis related gene expression *in vivo*. (**a**) Representative images of tumour samples obtained from nude mice injected with Huh7-2D, Huh7-3D, or Huh7/2% HUVEC-3D at 16 days after transplantation. (**b**,**c**) Tumour volumes were measured for 16 days after transplantation (**b**), tumour mass was determined at 16 days after transplantation using excised tumours (**c**). (**d**) H&E staining and IHC of endothelial specific markers (CD31, SMA, and endoglin) and cancer stem cell markers (CD24, CD133, and EpCAM) of tissue samples obtained from nude mice injected with Huh7-2D, Huh7-3D, or Huh7/2% HUVEC-3D. Scale bars, 200 μm. (**e**) Heat map of microarray data for EMT and angiogenesis genes in Huh7-2D, Huh7-3D, and Huh7/2% HUVEC-3D. (**f**) Gene ontology analysis of 1032 genes differentially expressed in tumour from nude mice injected Huh7/2% HUVEC-3D compared to Huh7-2D. *P < 0.05; **P < 0.01.

To evaluate the effect of 2% HUVECs in Huh7 spheroid tumourigenicity *in vivo*, we subcutaneously transplanted Huh7-2D cells, Huh7-3D, and Huh7/2% HUVECs-3D in nude mice and evaluated tumour growth over time. As expected, the growth of Huh7/2% HUVECs-3D was significantly increased; however, Huh7-3D did not show significant changes in growth rate as compared to Huh7-2D cells (Fig. 5a–c). However, haematoxylin and eosin (H&E) staining revealed histological changes in both Huh7-3D and Huh7/2% HUVECs-3D (Fig. 5d, left panel). More vascular morphologies and loose tight junctions were observed in Huh7-3D and Huh7/2% HUVECs-3D than in Huh7-2D. Particularly, mature and large vessels were observed in the Huh7/2% HUVEC-3D (Fig. 5d). Assessment of the expression of the vessel markers endoglin, CD31, and SMA demonstrated that HUVECs strongly promoted angiogenesis in the spheroids (Fig. 5d). The existence of HUVECs in the spheroids grown with 2% HUVECs was validated by RT-PCR using human *CD31*-specific primers (Supplementary Fig. S11). Several cancer stem cell markers (CD24, CD133, and EpCAM) were highly expressed in Huh7/2% HUVECs-3D (Fig. 5d).

Collectively, the results suggested that HUVECs more easily enhanced vascular and aggressive oncogenic properties *in vivo* than *in vitro*. To test this notion further, we performed a microarray analysis of the cell models *in vivo*. We identified 1,037 transcripts with a corrected *P*-value of 0.05 and at least a 50% change in expression in tumour mass from injected Huh7-3D and Huh7/2% HUVECs-3D as compared to tumour derived from 2D-cultured cells (Supplementary Fig. S12a). High-level pattern analysis using gene ontology tools highlighted the differential regulation of two key pathways: EMT and angiogenesis (Fig. 5e,f and Supplementary Table S4). As expected, 3D culture with HUVECs improved vascularization and aggressiveness via the expression of cancer stem cell markers *in vivo*. Interestingly, 2% HUVECs induced the production of mature vessels only *in vivo*. To figure out why, we analysed genes differentially expressed between Huh7-3D and Huh7/2% HUVECs-3D. Only 254 genes were significantly differentially expressed in tumours derived from Huh7/2% HUVECs-3D as compared to tumours of Huh7-3D, and these genes were largely related to fatty acid homeostasis and Wnt signalling (Supplementary Fig. S12b,c). Taken together, these data suggested that HUVECs induce cytokine signalling more effectively *in vivo* than *in vitro*, and they activate the EMT and angiogenesis pathways to induce tumour maturation *in vivo*. Based on the aggressive tumorigenic properties of the Huh7 spheroids cultured with 2% HUVECs, we considered them tumouroids; in what follows, we therefore use this term.

### Tumouroids survive at higher concentrations of anti-cancer drug than monolayer cells

**Figure 6:**
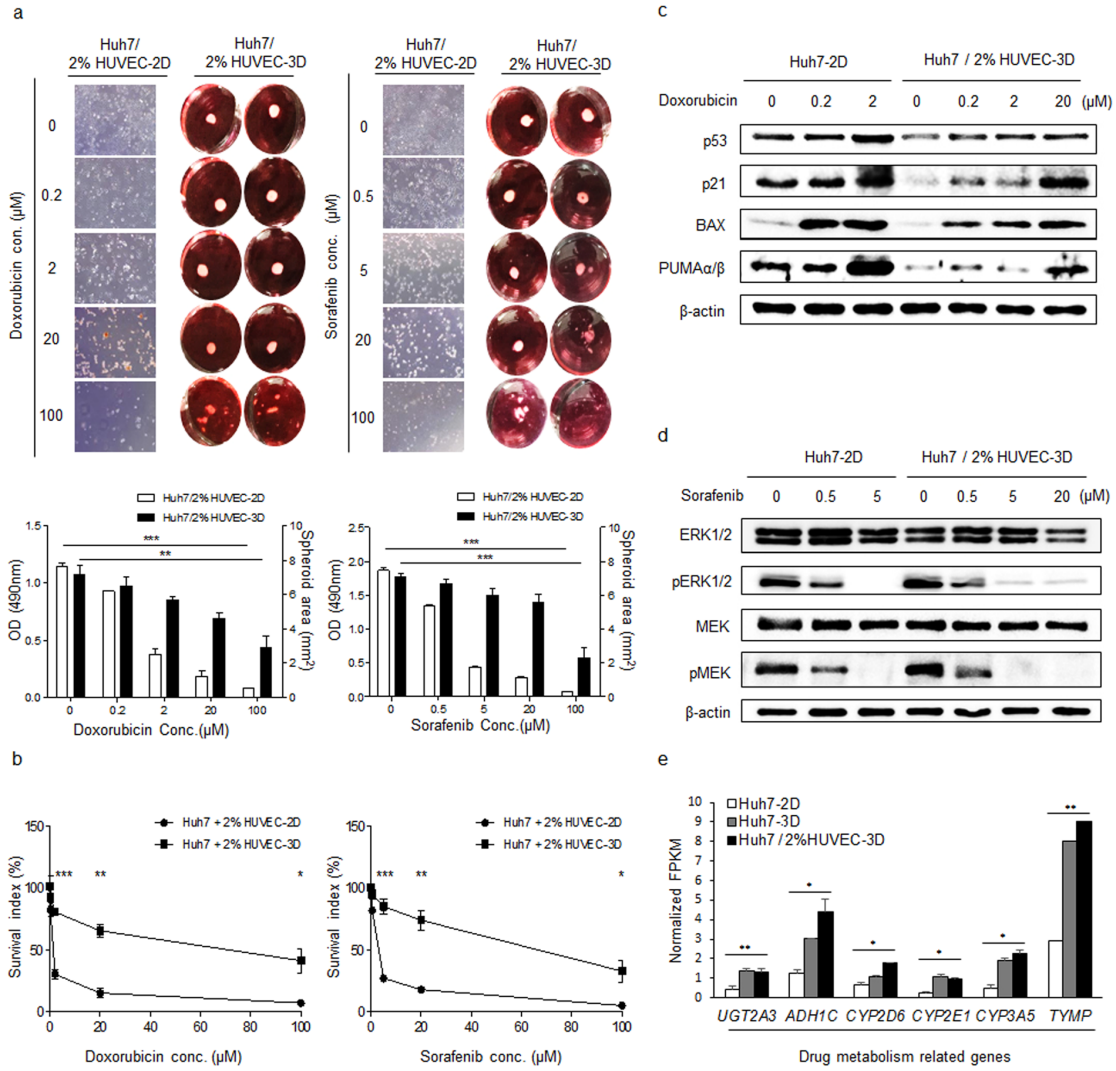
Tumouroids appear more resistant than 2D in anti-cancer drug tests. (**a**) Representative pictures (top) and quantitative graph (bottom) of Huh7/2% HUVEC-2D and Huh7/2% HUVEC-3D after 48 h of treatment with doxorubicin or sorafenib at various concentrations (0.5, 5, 20, and 100 μM) *in vitro.* (**b**) Percentage of inhibition of Huh7/2% HUVEC-2D and Huh7/2% HUVEC-3D after 48-h treatment with the indicated concentrations of doxorubicin and sorafenib. (**c**,**d**) Western blot analysis of doxorubicin (**c**) and sorafenib (**d**) target protein expression. (**e**) RNA expression levels of the indicated drug-metabolic genes in Huh7-2D, Huh7-3D and Huh7/2% HUVEC-3D. *P < 0.05; **P < 0.01; ***P < 0.001.

To assess drug concentration ranges applicable for drug validation assays using the SFU-based tumouroid, we performed anti-cancer drug tests using doxorubicin and sorafenib at various concentrations (0.2, 2, 20, and 100 μM doxorubicin and 0.5, 5, 20, and 100 μM sorafenib). Tumouroids and 2D cells were exposed to each drug for 48 h *in vitro*. Although we could macroscopically observe size decreases in our tumouroid model, we used microscopy to determine the proliferation of 2D cells (Fig. 6a). For 2D cells, we used a conventional GI_50_ (the concentration of drug causing a 50% reduction in cancer cell proliferation) assay using WST-1 reagent. The GI_50_ of doxorubicin and sorafenib in 2D cells was about 2 μM and 5 μM, respectively, while in the tumouroid, it was >20 μM for both drugs (Fig. 6b). These data indicated that the tumouroid is more resistant to both anti-cancer agents and thus, that it allows drug testing at wider concentration ranges than the 2D model. Western blotting to detect changes in the expression of the drug target molecules indicated that doxorubicin induced p53 activation and sorafenib inhibited pERK in both models (Fig. 6c,d). However, in 2D culture, target expression analysis was hampered by the fact that most cells died after treatment with each of both drugs at 20 μM. Transcripts related to drug metabolism induced the tumouroid character. In fact, the levels of key regulators of drug metabolism were higher in tumouroids than in 2D cells. Especially, CYP2D6 expression was significantly higher in spheroids cultured with HUVECs than in those cultured without (Fig. 6e). These data supported that tumouroids represent an accurate model for anti-cancer testing at a broad range of concentrations.

To allow comparison of our *in vitro* tumouroid model with *in vivo* HCC models for drug testing, we used a conventional *in vivo* xenograft assay in mice that were orally administrated doxorubicin at 5 mg per kilogram (mpk) or sorafenib at 30 mpk. Tumour size was measured for 7 to 8 days. doxorubicin was effective ( >50% inhibition) at the given dose of 5 mpk, and sorafenib was effective at the given dose of 30 mpk, for a 20-g mouse (Supplementary Fig. S13). Higher drug concentrations were required for the tumouroid and *in vivo* xenograft models than for the 2D model, which can be explained by differences in cell–cell interaction and gradients in the cellular conditions. Thus, we attempted to provide an *in vitro* tumour model that narrowed the gap between *in vitro* and *in vivo* models for more reliable anti-cancer drug testing.

## Discussion

Various types of 3D culture systems have been well established, using different devices or materials used for generating the 3D architecture^12, 13^. As the traditional hanging-drop method is relatively simple and has shown high reproducibility for numerous cell lines, it is widely used for generating cell spheroids. However, potential drawbacks are the limited volume of the cell suspension droplet and the difficulty in changing the culture medium. A second conventional 3D culture method uses scaffolds such as nanoparticles and hydrogels, which support 3D architectures. However, separating the cells from such materials is relatively complicated. The last method is agitation culture; however, this approach requires complicated and relative expensive instruments such as cell spinner flask systems or rotary cell culture systems. While current spheroid models have provided insights into the mechanisms of tumourigenicity, to overcome some of the inconveniences of current 3D cell culture methods, we developed the SFU, composed of a universal 15-ml tube with a filter cap. By combining the hanging-drop and rotation culture methods, we were able to produce large (>3 mm), homogenous Huh7 spheroids. Additionally, the SFU allowed size control of the spheroids. One advantage of the large spheroids is that changes can be clearly observed by naked eye in anti-cancer drug tests. It could be compared that macroscopic measurement is allow in mouse xenograft assays for observation of the tumour regression. Additionally, the large spheroid could mimics some of the complex tumour microenvironment, including hypoxia. Tumour tissues are normoxic near blood vessels and gradually become hypoxic with increasing distance from the vessels. Hypoxia plays a critical role in tumour progression by regulating ECM and angiogenesis^12^. Compare to human liver cancer tissues, the large-size Huh7 spheroid had a hypoxic core and a highly proliferative surface since it is surrounded by culture medium. Thus the core, relatively far from medium, had the hypoxic condition, but the surface, contacted to medium always, had proliferative cells, which might activated ERK signalling which plays a critical role in tumour cell proliferation in 3D culture conditions. Our data supported previous reports on the regulatory role of pERK in other types of cancer cell spheroids^13^.

In the liver, complex interactions and signal exchange with surrounding endothelial cells and fibroblasts are crucial to hepatic cell metabolic functionality and survival under toxic conditions^14^. Previous studies have indicated that hepatocytes cultured on fibroblasts show increased albumin and urea production and retain hepatocyte morphology^15–17^. Fibroblasts can provide ECM molecules, which can be of importance in cell–cell contacts for survival and proliferation of the hepatocytes^18, 19^. However, we observed only limited improvement of proliferation, survival, and functionality when spheroids were co-cultured with fibroblasts. It has been reported that fibroblasts secrete TGFβ, which can delay cancer cell proliferation^20^. In contrast, HUVECs improved the cell viability and evaded apoptosis, which aided in producing spheroids of relevant sizes (3–4 mm). Spheroid architecture can help in enhancing biological characteristics to mimic tumour tissues^21^. Previous studies have shown that co-culture of tumours with endothelial cells causes reprograming of tumour signalling, resulting in a phenotypic switch that includes growth, migration, activation, and morphogenic changes such as neovasculature^22^.

We optimized the conditions of co-culture with HUVECs to produce a Huh7 spheroid that effectively mimics human liver tumour. Interestingly, of all concentrations tested, 2% HUVECs improved the tumourigenicity of Huh7 spheroid the most effectively. It promoted HCC-related gene expression and furthermore, increased the expression of cancer stem cell markers including CD133, EpCAM, and CD 24, with CD133 being the most abundant in the Huh7 spheroids. Accordingly, we concluded that HUVECs confer aggressiveness to the spheroid. These data support a report by Takai *et al*., who showed that EpCAM is a major factor improving the tumourigenicity of Huh1 in 3D conditions^23^. However, we speculate that high concentrations of HUVECs in Huh7 spheroids negatively affect cell–cell connections between Huh7 cells, as HUVECs alone did not form spheroids and died early during spheroid formation. Therefore, 2% of HUVECs were selected as an optimum for the SFU system without any scaffold for 3D cell culture. RNA-seq data showed that Huh7 spheroids co-cultured with HUVECs produced expression patterns similar to those in *in vivo* tumour, with up-regulated drug metabolism and down-regulated glycan biosynthesis and metabolism. They recapitulate cell–cell/matrix interactions between cancer cells and the microenvironment, as well as transport properties^24, 25^. Moreover, HUVECs activated gene expression of MAPK signalling, this result is consistent with increasing pERK protein level of Huh7-3D.

HUVECs effectively regulated the cell signalling involved in cell survival, proliferation, migration, and invasion in the Huh7 spheroid. However, the concentration used did not suffice to generate tight cell–cell interactions. Therefore, we hypothesize that extracellular signalling of HUVECs, such as cytokine-mediated signalling, supported Huh7 cell viability. To test the contribution of extracellular signalling, we fabricated a device, composed of a permeable inner dish and an outer dish, to observe the effects of exchange of extracellular molecules. The results indicated that VEGF and angiogenin secreted by the HUVECs played a role in the induction of Huh7 proliferation and invasion. These cytokines activated receptor tyrosine kinases; therefore, MAPK signalling by HUVECs must have been implicated in the survival of the Huh7 spheroids.

Although 2% of HUVECs was not sufficient to configure vessel architecture *in vitro*, it promoted the generation and maturation of vessels in Huh7 spheroids *in vivo*. Moreover, this concentration of HUVECs promoted cancer stem cell marker expression, which is associated with tumour progression^25^, *in vivo*. Thus, HUVECs contributed to vascularization and aggressiveness of the Huh7 spheroid. We observed differential regulation of two key pathways—EMT and angiogenesis—in Huh7 spheroids as compared to Huh7-2D *in vivo*. However, there were no significant differences between Huh7-3D and Huh7/2% HUVECs-3D. We speculate that the 3D architecture contributed to the improved oncogenic properties *in vivo*. A small portion of genes related to fatty acid homeostasis and Wnt signalling pathways were expressed differentially in Huh7 spheroids grown with as compared to those grown without 2% HUVECs. Future studies will need to determine key pathways or factors to inducing tumour vascularization *in vivo* and translate these into Huh7 spheroids *in vitro*.

Our study has several important implications for *in vitro* tumour models. We report an optimized method for generating a tumouroid of sufficiently large size that enables macroscopic observation of tumour size changes after anti-cancer drug treatments and in which apoptotic and survival cell fates co-exist. Spheroids less than 200 μm have been used for drug testing and may be sufficient to recapitulate cell–cell or cell–matrix interaction, but they are not large enough to recapitulate oxygen gradients with hypoxic regions or proliferation gradients^26^. We also demonstrated by RNA-seq that the spheroid co-cultured with HUVECs in our culture system mimics *in vivo* tumour by regulating gene expression of drug metabolism and cytokine-mediated pathways. Our tumouroid did not develop vessel architecture *in vitro*; however, HUVECs in the spheroids contributed to form mature vasculature *in vivo* by activating angiogenesis and EMT. Moreover, they were more resistant to drug treatments than cells in 2D culture and could recapitulate the drug resistance observed in solid tumours^27^. We confirmed that endothelial cells contribute to tumourigenicity of HCC through molecular cross-talk between cells^28^. This finding could aid in improving the viability of patient-derived tumour *in vitro*, which to date has impeded the generation of patient-derived xenograft models and consequently, their clinical application. As the need for *in vitro* models that reflect *in vivo* conditions is increasing, we expect our method to impact the generation of 3D tumouroid models.

## Methods

### Reagents and antibodies

DMSO (Mylan, Amsterdam, Netherlands), doxorubicin (Sigma, St. Louis, MO, USA) and sorafenib (Tocris Bioscience, Bristol, UK) were used for *in vivo*/*in vitro* drug treatment assays. PD98059 (Sigma Aldrich) and LY294002 (Sigma Aldrich) were used for *in vivo*/*in vitro* drug treatment assays. Antibodies for fibronectin, E-cadherin, PUMAα/β, Bnip3, MCL-1, p53, p21, BAX, AKT, pAKT, SNAI1, vimentin (Santa Cruz Technologies, Dallas, TX, USA; 1:1000), HIF-1α, ERK, pERK, MEK, and pMEK (Cell Signaling Technologies, Danvers, MA, USA; 1:1000) were used for western blotting. β-actin (Sigma; 1:5000) served as loading control. CD24 (Santa Cruz Technologies; 1:100), HIF-1α, Ki-67, cleaved caspase-3, β-catenin, EpCAM (Cell Signaling Technologies; 1:100), CD133 (Miltenyi Biotec, Bergisch Gladbach, Germany; 1:100), CD31, endoglin (Abcam, Cambridge, MA, USA; 1:100) and SMA (Sigma, 1: 400) were used for IHC/ICC. VEGF (Santa Cruz Technologies) and angiogenin (eBioscience, San Diego, USA) were used for blocking assay at indicated concentrations.

### Cell lines and human cancer tissues

Huh7, Hep3B, HepG2, SK-Hep1, and MRC5 cells were obtained from the American Type Culture Collection (Rockville, MD, USA). T0056 cells were purchased from Applied Biological Materials Inc. (Richmond, BC, Canada). CK-K1 cells were kindly provided by Dr. Dae-Ghon Kim (Chonbuk National University Medical School and Hospital, Jeonju, Republic of Korea). Cells were cultured in high-glucose Dulbecco’s Modified Eagle’s Medium (DMEM) with 10% foetal bovine serum, 1% penicillin-streptomycin, and 1% l-glutamine. HUVECs were purchased from Lonza (Basel, Switzerland) and GFP-tagged HUVECs were purchased from Angio-Proteomie (Boston, MA, USA). HUVECs were cultured in EGM-2 medium (Lonza). All cells were grown in a humidified incubator at 37 °C with 5% CO_2_.

Primary HCC and normal liver tissues were obtained from patients who underwent surgery for HCC at Chonbuk National University Hospital (Jeonju, South Korea) in accordance with relevant guidelines and regulations. Informed consent was obtained from patients for the use of specimens for research purpose only. All experimental protocols were approved by the Institutional Ethics Committee/IRB of the Korea research institute of bioscience and biotechnology (KRIBB).

### Generation and culture of cell spheroids with/without HUVECs, T0056, or MRC5 cells

We designed an SFU composed of a conical tube with a filter-loaded cap, which was produced by SPL Science (Pocheon, Korea). Cell spheroids were generated using the hanging drop method and the SFU. Cells at various at various proportions were mixed and suspended in DMEM; 50-μl droplets with a density of 10^4^ cells μl^–1^ were used. The droplets were deposited on the inverted lid of a culture dish after which the lid was placed onto the PBS-filled dish; the cells were incubated at 37 °C for 48 h. Cell sheets or aggregates were transferred to the SFU or a 6-well plate for stationary culture and incubated in a rotary incubator at 37 °C for 72 h. To prepare co-culture spheroids, the cells were mixed at the indicated ratios before generating the hanging drops.

### Enzyme-linked immunosorbent assay (ELISA)

Supernatants were collected and stored at −70 °C until processing. Albumin, AFP, and VEGF secreted in the culture medium were measured by standard sandwich ELISA using human AFP and human VEGF ELISA kits (R&D systems, Minneapolis, MN, USA), according to the manufacturers’ instructions.

### Western blot analysis

Cells were lysed in RIPA buffer (50 mM Tris-HCl, pH 8.0, 150 mM NaCl, 5 mM EDTA, and 0.1% SDS) and the cell debris was cleared by centrifugation at 15,000 × *g* for 10 min. The lysates were boiled in SDS sample buffer for 5 min. The proteins were resolved by SDS-PAGE and transferred to a PVDF membrane (Millipore, Billerica, MA, USA). The membranes were blocked in 5% skim milk in PBST (0.5% Tween-20 in PBS) at room temperature (RT) for 1 h. The membranes were incubated with the appropriate antibodies in PBST for 1 h at RT or overnight at 4 °C and then with secondary antibody in PBST for 1 h at RT. The proteins were detected with a chemiluminescence kit (Intron Biotech, Seoul, Korea and Millipore).

### Live/dead cell staining

Cell viability of the spheroids was examined with a two-colour live/dead cell assay kit (Invitrogen, Waltham, MA, USA). Calcein-AM (2 μM) and EthD-1 (4 μM) were added and incubated for 15 min at 37 °C with 10% CO_2_. After washing with PBS, the cells were visualized under a fluorescence microscope (Panasonic, Kadoma, Osaka, Japan). Calcein and EthD-1 were excited using a green fluorescence filter (485 ± 10 nm) and a red fluorescence filter (530 ± 12.5 nm), respectively.

### Immunohistofluorescence and immunocytochemical analysis of the spheroids

Cells embedded in 2% agar or graft-bearing tumours were removed from nude mice and were fixed with 10% formalin, embedded in paraffin, and cut at 5-μm thickness, followed by permeabilisation with 0.5% Triton X-100 in PBS (PBST) for 10 min. For antigen retrieval, tissue sections immersed in 10 mM citrate buffer solution (pH 6.0) were placed in a microwave for 20 min. The sections were incubated in 2% BSA with 0.2% fish-skin gelatin at RT for 1 h to block non-specific binding and the sections were incubated with primary antibodies overnight at 4 °C. Staining was visualized using peroxidase-conjugated antibodies to mouse immunoglobulin using the Vectastain Elite kit and 3,3-diaminobenzidine (DAB). Nuclei were counterstained with haematoxylin.

### RT-qPCR

Total RNA was isolated using a TRIzol reagent-based kit (Intron Biotech). Reverse transcription was performed using SuperScript III First-Strand Synthesis System for RT-PCR (Thermo Fisher Scientific, Waltham, MA, USA) according to the manufacturer’s protocol. cDNA was amplified with specific primers and SYBR Premix Ex Taq (Takara Bio, Otsu, Shiga, Japan and Agilent Technologies, Santa Clara, CA, USA). The target mRNA levels were presented relative to the amount of beta tubulin mRNA. qPCR was performed according to the manufacturer’s instructions (Applied Biosystems, Waltham, MA, USA and Agilent Technologies). The primer sequences are listed in Supplementary Table S1.

### Flow cytometry

Cell spheroids were re-suspended in TE buffer for 5 min and washed with PBS containing 1% FBS. After washing, flow cytometry was performed on a BD FACS Canto II and data were analysed with FlowJo software.

### RNA-seq

Total RNA was extracted from tissue samples using the RNeasy Mini Kit (Qiagen, Hilden, Germany) according to the manufacturer’s instructions. The RNA was analysed on a 2100 Bioanalyzer (Agilent Biotechnologies, Palo Alto, USA) with the RNA 6000 Nano Labchip kit. Only RNA samples of high quality (RNA integrity number ≥7.5) were used for sequencing. Approximately 0.5–4 μg of total RNA was used for library construction using the TruSeq RNA Sample Preparation Kit V2. The libraries were clustered using HiSeq Rapid Cluster Kit V2 with Flow Cell V2 and HiSeq rapid SBS V2. Samples were sequenced on a HiSeq. 2500 machine (Illumina, San Diego, CA, USA) using the standard Illumina RNA-seq protocol, with a read length of 2 × 100 bases. Ten samples were multiplexed 10 per lane, and sequencing produced an average of 19 million mappable read pairs per sample.

### Read mapping and quantification

The quality of obtained raw reads was first examined using the software package FastQC. The reads were trimmed for low-quality ends using a phred quality threshold score of 20. Adapters were trimmed using cutadapt and sickle. Reads with a trimmed length below 50 bp were dropped. After filtering for sequencing errors, the processed reads were mapped to the reference genome using TotHat v2.1.0 (based on Bowtie2 v2.1.0), with default parameter settings. The human reference transcriptome annotation and reference genome from hg19 were used, covering 25,370 unique genes, downloaded from iGenome. On average 94% of reads aligned in at least one region of the reference genome for all samples (Supplementary Table S2). To obtain quantification scores for all human genes and transcripts, FPKM (fragments per kilobase of exon model per million mapped reads) values were calculated using Cufflinks v2.2.1, which corrects for transcript length and the total number of mapped reads from the library to compensate for different read depths for different samples. To normalize library size, cuffnorm was used.

### Collection of conditioned medium (CM) from HUVECs and antibody blocking assay

To determine the effect of secreted proteins from HUVECs, Huh7 cells were co-cultured with/without HUVECs in a co-culture dish (SPL Life Sciences), provided with an insert mesh to allow bi-directional signal exchange but not transmigration. After 5 days, Huh7 cells were counted with a haemocytometer and stained with crystal violet. To assess the effects of the CM, HUVECs were seeded at 60–70% confluence in a 100-mm dish and cultured with DMEM containing 10% FBS for 48 h. The CM was filtered through a 0.45-μm filter. For the proliferation assay, Huh7 cells were cultured at 1 × 10^5^ cells with/without CM in 6-well plates. For the antibody-based blocking assay, VEGF- and EGF-blocking antibodies at indicated concentrations were treated with CM. After 5 days of culture, the cells were trypsinised and counted with a haemocytometer. Cell viability was assayed with crystal violet staining.

### Human angiogenesis array

The expression levels of angiogenesis-associated proteins were analysed using media from Huh7 cell spheroids cultured with or without 2% HUVECs using a human angiogenesis array kit (R&D Systems) according to the manufacturer’s instructions. Briefly, 500 μl of culture supernatants were mixed with a cocktail of biotinylated anti-angiogenic antibodies and incubated with the array membranes overnight at 4 °C. The membranes were washed and then incubated with HRP-conjugated streptavidin for 30 min at RT. Chemiluminescence was used for signal detection. Staining intensities were determined with the ImageJ software.

### Spheroid-based proliferation assay

To examine spheroid-based growth inhibition, Huh7-3D or Huh7/2% HUVECs-3D (5 × 10^5^ cells per spheroid) were added to each well of a 96-well round-bottomed plate. The spheroids were incubated with serum-free DMEM for 24 h. After starvation for 24 h, they were treated with serum-free DMEM containing VEGF-neutralizing antibody (5, 10, 30 µg/ml) or angiogenin-neutralizing antibody (5, 10, 20 µg/ml) for 72 h. To measure growth inhibition, images of spheroids were captured using a Nikon digital camera before adding antibody (0 h) and 72 h after antibody treatment (72 h). The spheroid areas were quantified using ImageJ at 0 h and 72 h. The cell viability of 3D spheroids was examined by the CellTiter-Glo^®^ assay (Promega, Wisconsin, USA). 3D spheroids were cultured and added to each well with CellTiter-Glo 3D reagent. After 30 min, the luminescence signal (representative of the amount of ATP generated by viable cells) was measured with a microplate reader. The spheroids were then collected and analysed by western blotting with indicated antibodies.

### Spheroid-based invasion assay

To examine spheroid-based invasion, Huh7-3D or Huh7/2% HUVEC-3D (5 × 10^5^ cells per spheroid) spheroids were embedded into Matrigel in a 24-well plate (a single spheroid/well). The spheroids were incubated with serum-free DMEM for 24 h. After starvation for 24 h, they were incubated with serum-free DMEM containing VEGF neutralizing antibody (30 µg/ml) or mouse IgG isotype control antibody (30 µg/ml) for 5 days. At 5 days after antibody treatment, invasiveness of the spheroids was measured under an inverted Olympus microscope (magnification 10×) and images were obtained. The sprouting areas per spheroid were quantified using ImageJ.

### *In vitro* anti-cancer efficacy test

Spheroids constructed in SFUs (size, 3–4 mm) were transferred into a 24-well plate and drugs were applied at the indicated concentrations in DMEM. Spheroid sizes were determined by measuring the surface area using ImageJ. To determine the GI_50_, Huh7 cells (5 × 10^3^ cells) were seeded into 96-well plates and incubated in DMEM containing serially diluted drugs for 48 h. Cell viability was measured using the CytoX cell viability assay kit (LPS Solution, Daejeon, Korea) per the manufacturer’s instructions.

### Animal experiments (tumourigenicity test and xenograft assay)

Seven-week-old female BALB/c nude mice (BALB/cSlc-nu/nu) were purchased from Japan SLC, Inc. All experimental protocols were approved by the Korea Research Institute of Bioscience and Biotechnology (KRIBB) Animal Care and Uses Committee. All of procedures were performed by accordance with the appropriate KRIBB biosafety guidelines and regulations. To test tumourigenicity *in vivo*, Huh7 monolayers (5 × 10^6^) and 3D Huh7 spheroids with/without HUVECs (5 × 10^6^) were transplanted subcutaneously. Tumour size was measured every second day for 16 days as reported previously^29, 30^. For the *in vivo* xenograft assay for anti-cancer drugs, Huh7 cells (1 × 10^7^) were transplanted subcutaneously. When the tumour size reached 60–70 mm^3^, sorafenib was orally administrated. Doxorubicin was intravenously injected when the tumour volume reached approximately 30 mm^3^. Drugs were administered daily for 7 days and tumour size was measured be measured be every second day as reported previously^29, 30^.

### Microarray analysis

Per sample, 300 ng of total RNA was used as input as recommended by manufacturer (http://www.affymetrix.com). Total RNA was converted to double-stranded cDNA using a random hexamer incorporating a T7 promoter. Amplified RNA (cRNA) was generated from the double-stranded cDNA template though an *in vitro* transcription reaction, and purified with the Affymetrix sample clean-up module. Single-stranded cDNA (ss-cDNA) was regenerated through a random-primed reverse transcription using a dNTP mix containing dUTP. The ss-cDNA was then fragmented using UDG and APE 1 restriction endonucleases and end-labelled by a terminal transferase reaction incorporating a biotinylated dideoxynucleotide. Fragmented end-labelled cDNA was hybridized to the GeneChip Human Gene 2.0 ST arrays for 16 h at 45 °C and at 60 rpm as described in the Gene Chip Whole Transcript (WT) Sense Target Labeling Assay manual (Affymetrix). After hybridization, the chips were stained using streptavidin–phycoerythrin conjugate and washed in a Genechip Fluidics Station 450 (Affymetrix).

### Data analysis

The array was scanned using Affymetrix Model 3000 G7 scanner and image data was extracted through Affymetrix Command Console software v1.1. The raw.cel file generated through above procedure including expression intensity data was used for the next step. Expression data were generated by Affymetrix Expression Console software version1.4. For normalization, the Robust Multi-Average algorithm implemented in Affymetrix Expression Console software was used.

### Statistical analysis

Data are displayed as the mean ± SEM and were analysed with Prism version 5.0 (GraphPad Software). Unpaired Student’s t-test was used for comparisons between two groups. We used one-way ANOVA with Tukey’s post hoc tests to compare multiple groups. A *p*-value < 0.05 was considered to be significant.

## Electronic supplementary material

Supplementary information
