# Supplementary material for: Cell Spheroids with Enhanced Aggressiveness to Mimic Human Liver Cancer In Vitro and In Vivo
Source: Sci Rep. 2017 Sep 5;7:10499. doi: 10.1038/s41598-017-10828-7 (PMC5585316; doi:10.1038/s41598-017-10828-7)
Supplement: Supplementary file 1 — Supplementary information [file 41598_2017_10828_MOESM1_ESM.pdf]

Cell Spheroids with Enhanced Aggressiveness to Mimic Human Liver Cancer *In Vitro* and *In Vivo*

Hong-Ryul Jung[1$], Hyun Mi Kang[1$], Jea-Woon Ryu[3], Dae-Soo Kim[2,3], Kyung Hee Noh [1], Eun-Su Kim[1,2], Ho-Joon Lee[3], Kyung-Sook Chung[2,3], Hyun-Soo Cho[2,3], Nam-Soon Kim[2,3], Dong-Soo Im[1], Jung Hwa Lim[1,*] & Cho-Rok Jung[1,2,*]

[1] Gene Therapy Research Unit, Korea Research Institute of Bioscience and Biotechnology, Daejeon, 305-333, Republic of Korea.

[2] University of Science and Technology, Daejeon, Republic of Korea.

[3] Genome Research Center, Korea Research Institute of Bioscience and Biotechnology, Daejeon, 305-333, Republic of Korea.

[$] These author contributed equally to this work.

*Correspondence and requests for materials should be addressed to C.R. J. (crjung@kribb.re.kr) or J.H. L (jhwa@kribb.re.kr).

Supplementary table S1. Primers used for real-time PCR

Gene Name	Direction	Sequence
human VEGF	Forward	5′-AGGGCAGAATCATCACGAAGT-3′
	Reverse	5′-AGGGTCTCGATTGGATGGCA-3′
human AFP	Forward	5′-CTTTGGGCTGCTCGCTATGA-3′
	Reverse	5′-GCATGTTGATTTAACAAGCTGCT-3′
human ALB	Forward	5′-GAGACCAGAGGTTGATGTGATG-3′
	Reverse	5′-AGTTCCGGGGCATAAAAGTAAG-3′
human CYP3A4	Forward	5′-AAGTCGCCTCGAAGATACACA-3′
	Reverse	5′-AAGGAGAGAACACTGCTCGTG-3′
human IGF2	Forward	5′-GACACCCTCCAGTTCGTCTG-3′
	Reverse	5′-CGGGGTATCTGGGGAAGTTG-3′
human MET	Forward	5′-GTTATACCCCAGCCCAAACCA-3′
	Reverse	5′-CCACCACTGGCAAAGCAAAAT A-3′
human RHOA	Forward	5′-TTCGTTGCCTGAGCAATGG-3′
	Reverse	5′-TGTGTCCCACAAAGCCAACT-3′
human TCF4	Forward	5′-ACCCCAGGACCCTTACAGAG-3′
	Reverse	5′-TGTCAGGTCCTCATCGTCAT-3′
human TNFSF10	Forward	5′-GGGACCCCAATGACGAAGAG-3′
	Reverse	5′-GTCCCAGTTATGTGAGCTGC-3′
human TGFA	Forward	5′-CTGAGTGCAGACCCGCC-3′
	Reverse	5′-GGTGATGGCCTGCTTCTTCT-3′
human CCNA1	Forward	5′-GATAACGACGGGAAGAGCGG-3′
	Reverse	5′-CTGCTGCTGGAAGACGAAATC-3′
human CCNB1	Forward	5′-GTGTAGGTCCTTGGCTGGTC-3′
	Reverse	5′-AGCTGTTCTTGGCCTCAGTC-3′
human CCND1	Forward	5′-GATGCCAACCTCCTCAACGA-3′
	Reverse	5′-GGAAGCGGTCCAGGTAGTTC-3′
human CCND2	Forward	5′-CCGACAACTCCATCAAGCCT-3′
	Reverse	5′-CATCGACGGTGGGTACATGG-3′
human CCNE1	Forward	5′-CCCCATCATGCCGAGGGA-3′
	Reverse	5′-GGGTCTGCACAGACTGCATTA-3′
human BAX	Forward	5′-CCCTTTTGCTTCAGGGTTTCA-3′
	Reverse	5′-GGAAAAAGACCTCTCGGGGG-3′
human MCL1	Forward	5′-TGCTTCGGAAACTGGACATCA-3′
	Reverse	5′-TAGCCACAAAGGCACCAAAAG-3′
human TUBB	Forward	5′-GTGGTACGGAAGGAGGTCGAT-3′
	Reverse	5′-GGCGGAACATGGCAGTGAAC-3′
human CD24	Forward	5′-CTCCTACCCACGCAGATTTATTC-3′
	Reverse	5′-AGAGTGAGACCACGAAGAGAC-3′
human CD44	Forward	5′-CTGCCGCTTTGCAGGTGTA-3′
	Reverse	5′-CATTGTGGGCAAGGTGCTATT-3′
human CD133	Forward	5′-AGTCGGAAACTGGCAGATAGC-3′
	Reverse	5′-GGTAGTGTTGTACTGGGCCAAT-3′
human EPCAM	Forward	5′-AATCGTCAATGCCAGTGTACTT-3′
	Reverse	5′-TCTCATCGCAGTCAGGATCATAA-3′
human CD31	Forward	5′-AACAGTGTTGACATGAAGAGCC-3′
	Reverse	5′-TGTAAAACAGCACGTCATCCTT-3′
mouse CD31	Forward	5′-AAAGGAGGTGACAGAAGGCG-3′
	Reverse	5′-CATCCCAGGGGGCTTGATTT-3′
human SNAI1	Forward	5′-ACTGCAACAAGGAATACCTCAG-3′
	Reverse	5′-GCACTGGTACTTCTTGACATCTG-3′
human VIMENTIN	Forward	5′-GACGCCATCAACACCGAGTT-3′
	Reverse	5′-CTTTGTCGTTGGTTAGCTGGT-3′
human CTNNB1	Forward	5′-AAAGCGGCTGTTAGTCACTGG-3′
	Reverse	5′-CGAGTCATTGCATACTGTCCAT-3′
human/mouse GAPDH	Forward	5′-ATGGGGAAGGTGAAGGTCGG-3′
	Reverse	5′-TGGTTCACACCCATGACGAA-3′

Supplementary table S2. Up-regulated gene list in Huh7 / 2% HUVEC-3D compare to Huh7-3D

General Gene Symbol	Gene Name	Fold Change
TFF2	trefoil factor 2	3.78812785
NOX1	NADPH oxidase 1	3.48305528
AGXT	alanine-glyoxylate aminotransferase	3.0051203
TMEM88	transmembrane protein 88	2.18647159
S100A9	S100 calcium binding protein A9	2.18216233
LOC101928292	uncharacterized LOC101928292	2.15120519
DNAJC28	DnaJ heat shock protein family (Hsp40) member C28	2.11336184
NAT2	N-acetyltransferase 2	2.05416065
SLC25A48	solute carrier family 25 member 48	2.04612704
CA4	carbonic anhydrase 4	2.03456296
EGR1	early growth response 1	2.03308999
GLYATL1	glycine-N-acyltransferase like 1	1.9993384
FAM45B	family with sequence similarity 45 member B, pseudogene	1.99922857
LINC00266-1	long intergenic non-protein coding RNA 266-1	1.9936374
CTD-3080P12.3	uncharacterized LOC101928857	1.98398727
TRIM50	tripartite motif containing 50	1.97585069
GDAP1L1	ganglioside induced differentiation associated protein 1 like 1	1.95566223
TFF3	trefoil factor 3	1.95372454
DGKK	diacylglycerol kinase kappa	1.85831815
TTR	transthyretin	1.84545284
CHI3L1	chitinase 3 like 1	1.84346764
PRR22	proline rich 22	1.83383261
CYP2D7P	cytochrome P450 family 2 subfamily D member 7	1.83192216
LOC100133445	uncharacterized LOC100133445	1.82650515
SHROOM1	shroom family member 1	1.80881376
CXCL3	C-X-C motif chemokine ligand 3	1.80757469
C6orf222	chromosome 6 open reading frame 222	1.79077324
SPATC1	spermatogenesis and centriole associated 1	1.78041041
LRRC39	leucine rich repeat containing 39	1.75971013
CAPN3	calpain 3	1.75316902
LOC389332	uncharacterized LOC389332	1.73839749
UGT1A1	UDP glucuronosyltransferase family 1 member A1	1.73521994
PROZ	protein Z, vitamin K dependent plasma glycoprotein	1.71104623
LOC101927755	uncharacterized LOC101927755	1.70890579
MVD	mevalonate diphosphate decarboxylase	1.69795336
PROC	protein C, inactivator of coagulation factors Va and VIIIa	1.6816193
CXCL2	C-X-C motif chemokine ligand 2	1.67124613
HCG4B	HLA complex group 4B (non-protein coding)	1.67100679
OCA2	OCA2 melanosomal transmembrane protein	1.66537139
OLFM3	olfactomedin 3	1.65176535
CBX3P2	chromobox 3 pseudogene 2	1.65126667
LINC00324	long intergenic non-protein coding RNA 324	1.64926551
UBE2Q2P1	ubiquitin conjugating enzyme E2 Q2 pseudogene 1	1.64498068
ALDH8A1	aldehyde dehydrogenase 8 family member A1	1.64343926
PYY2	peptide YY 2 (pseudogene)	1.64206406
AIM1L	absent in melanoma 1-like	1.63364201
TRIM74	tripartite motif containing 74	1.63282035
FJX1	four jointed box 1	1.62892879
SLC25A18	solute carrier family 25 member 18	1.62409296
CYP2D6	cytochrome P450 family 2 subfamily D member 6	1.61783227
ENTPD8	ectonucleoside triphosphate diphosphohydrolase 8	1.61474872
PLIN5	perilipin 5	1.61260629
EREG	epiregulin	1.61128876
EXOC3L4	exocyst complex component 3 like 4	1.61014975
MVK	mevalonate kinase	1.60013643
CLUHP3	clustered mitochondria homolog pseudogene 3	1.59930542
LETM2	leucine zipper and EF-hand containing transmembrane protein 2	1.59890994
BBC3	BCL2 binding component 3	1.59836535
RND1	Rho family GTPase 1	1.5975348
FAM43A	family with sequence similarity 43 member A	1.59470118
NAT8	N-acetyltransferase 8 (putative)	1.57909654
NDUFA6-AS1	NDUFA6 antisense RNA 1 (head to head)	1.57864847
GDF15	growth differentiation factor 15	1.57542222
FDPS	farnesyl diphosphate synthase	1.57534135
IBSP	integrin binding sialoprotein	1.57137019
MLXIPL	MLX interacting protein like	1.57121739
FOXN4	forkhead box N4	1.57066652
LSS	lanosterol synthase (2,3-oxidosqualene-lanosterol cyclase)	1.57066234
NEURL3	neuralized E3 ubiquitin protein ligase 3	1.56936384
F12	coagulation factor XII	1.56912079
HP	haptoglobin	1.56800913
FGF21	fibroblast growth factor 21	1.56655243
TAT	tyrosine aminotransferase	1.56529062
CLDN3	claudin 3	1.56519957
ITIH4	inter-alpha-trypsin inhibitor heavy chain family member 4	1.56420574
AP1M2	adaptor related protein complex 1 mu 2 subunit	1.56417128
SGPP2	sphingosine-1-phosphate phosphatase 2	1.56250426
G0S2	G0/G1 switch 2	1.56036951
NRG4	neuregulin 4	1.5582626
CCDC154	coiled-coil domain containing 154	1.55429922
LBP	lipopolysaccharide binding protein	1.55311307
RCN3	reticulocalbin 3	1.55217691
CARD9	caspase recruitment domain family member 9	1.54904574
LYPD6B	LY6/PLAUR domain containing 6B	1.54421954
ZNF596	zinc finger protein 596	1.54131097
LOC100288162	uncharacterized LOC100288162	1.54107637
LINC01004	long intergenic non-protein coding RNA 1004	1.5324401
GRASP	general receptor for phosphoinositides 1 associated scaffold protein	1.53203381
DGCR10	DiGeorge syndrome critical region gene 10 (non-protein coding)	1.52861838
LCN12	lipocalin 12	1.52756605
PTK6	protein tyrosine kinase 6	1.52465729
SH2D5	SH2 domain containing 5	1.51876123
CNTD2	cyclin N-terminal domain containing 2	1.51740611
LPXN	leupaxin	1.51658888
PDX1	pancreatic and duodenal homeobox 1	1.51590289
CSF3	colony stimulating factor 3	1.5143814
LTB	lymphotoxin beta	1.51365863
NR0B2	nuclear receptor subfamily 0 group B member 2	1.51063568
VNN3	vanin 3	1.51014959
DAO	D-amino acid oxidase	1.50999231
DOCK5	dedicator of cytokinesis 5	1.50805753
MIR22HG	MIR22 host gene	1.50772112
RRN3P3	RRN3 homolog, RNA polymerase I transcription factor pseudogene 3	1.5071051
TFF1	trefoil factor 1	1.50548113
FOXH1	forkhead box H1	1.50300377
PDE4B	phosphodiesterase 4B	1.50249727

Supplementary table S3. Down-regulated gene list in Huh7 / 2% HUVEC-3D compare to Huh7-3D

General Gene Symbol	Gene Name	Fold Change
NXPE3	neurexophilin and PC-esterase domain family member 3	0.66581325
DPP4	dipeptidyl peptidase 4	0.6656904
ERMP1	endoplasmic reticulum metallopeptidase 1	0.66191089
SLC41A1	solute carrier family 41 member 1	0.65933496
KLRC2	killer cell lectin like receptor C2	0.65872608
TRIM71	tripartite motif containing 71	0.65809878
INHA	inhibin alpha subunit	0.65788884
LAMB2P1	laminin subunit beta 2 pseudogene 1	0.6566416
AZGP1	alpha-2-glycoprotein 1, zinc-binding	0.6558765
NKD1	naked cuticle homolog 1	0.6535103
GMCL1	germ cell-less, spermatogenesis associated 1	0.6522964
TMEM74	transmembrane protein 74	0.65083633
GBP1	guanylate binding protein 1	0.64957168
MATN3	matrilin 3	0.64923643
TLR7	toll like receptor 7	0.64889971
PALM3	paralemmin 3	0.6469237
PCDH20	protocadherin 20	0.64632995
C1orf21	chromosome 1 open reading frame 21	0.6416627
ABCA3	ATP binding cassette subfamily A member 3	0.64149744
NOTCH3	notch 3	0.63740387
TTPA	alpha tocopherol transfer protein	0.63738974
KLHL14	kelch like family member 14	0.63714516
ENTPD7	ectonucleoside triphosphate diphosphohydrolase 7	0.63685097
CRLF1	cytokine receptor like factor 1	0.63635736
PCAT6	prostate cancer associated transcript 6 (non-protein coding)	0.63503527
IL15RA	interleukin 15 receptor subunit alpha	0.63455012
ELK3	ELK3, ETS transcription factor	0.62982305
MAP1A	microtubule associated protein 1A	0.62970311
TMEM59L	transmembrane protein 59 like	0.62963477
ITPRIPL1	inositol 1,4,5-trisphosphate receptor interacting protein-like 1	0.62751063
HSPA8	heat shock protein family A (Hsp70) member 8	0.62184231
P2RY6	pyrimidinergic receptor P2Y6	0.61127615
KCNJ8	potassium voltage-gated channel subfamily J member 8	0.60793169
HSPH1	heat shock protein family H (Hsp110) member 1	0.60788419
SRGAP2D	SLIT-ROBO Rho GTPase activating protein 2D (pseudogene)	0.60721572
AP1S2	adaptor related protein complex 1 sigma 2 subunit	0.60588306
EEF1A2	eukaryotic translation elongation factor 1 alpha 2	0.60561802
HIP1	huntingtin interacting protein 1	0.59787546
CYP26A1	cytochrome P450 family 26 subfamily A member 1	0.5967567
MT1X	metallothionein 1X	0.59659777
OLFML2A	olfactomedin like 2A	0.59625253
HLA-B	major histocompatibility complex, class I, B	0.59338255
RARG	retinoic acid receptor gamma	0.59287146
RAB42	RAB42, member RAS oncogene family	0.59039646
HLA-C	major histocompatibility complex, class I, C	0.58861312
SEC24D	SEC24 homolog D, COPII coat complex component	0.57440357
MT1A	metallothionein 1A	0.57382541
ADAMTS12	ADAM metallopeptidase with thrombospondin type 1 motif 12	0.5685699
PRKAR2A	protein kinase cAMP-dependent type II regulatory subunit alpha	0.56745358
S1PR3	sphingosine-1-phosphate receptor 3	0.56609567
BHLHA15	basic helix-loop-helix family member a15	0.56579826
C12orf36	long intergenic non-protein coding RNA 1559	0.56008945
LEFTY2	left-right determination factor 2	0.55923048
COL1A2	collagen type I alpha 2 chain	0.55802553
UGT2B15	UDP glucuronosyltransferase family 2 member B15	0.55593416
LBH	limb bud and heart development	0.55462934
ZC3HAV1L	zinc finger CCCH-type containing, antiviral 1 like	0.55422443
PDK4	pyruvate dehydrogenase kinase 4	0.55015829
EXOC6B	exocyst complex component 6B	0.54765979
HES7	hes family bHLH transcription factor 7	0.54551651
LNPEP	leucyl and cystinyl aminopeptidase	0.54302851
CYBRD1	cytochrome b reductase 1	0.54118618
ULBP3	UL16 binding protein 3	0.53978992
RAET1E-AS1	RAET1E antisense RNA 1	0.53942951
DOC2B	double C2 domain beta	0.53686146
COL11A1	collagen type XI alpha 1 chain	0.53548969
ZP3	zona pellucida glycoprotein 3	0.53184531
PALM2-AKAP2	PALM2-AKAP2 readthrough	0.52904004
TMEM27	transmembrane protein 27	0.50194115
MT2A	metallothionein 2A	0.50162635
CRABP2	cellular retinoic acid binding protein 2	0.49839005
IFIT1	interferon induced protein with tetratricopeptide repeats 1	0.48931506
FDCSP	follicular dendritic cell secreted protein	0.48645052
FLNC	filamin C	0.48137853
HSPA6	heat shock protein family A (Hsp70) member 6	0.31028007
TAC3	tachykinin 3	0.26062531

Supplementary table S4. Up-regulated EMT and angiogenesis related gene list in Huh7-3D and Huh7 / 2% HUVEC-3D *in vivo*

Official Gene Symbol	Gene Name	Official Gene Symbol	Gene Name
APOH	apolipoprotein H	BMP1	bone morphogenetic protein 1
APOB	apolipoprotein B	SLC12A6	solute carrier family 12 member 6
GPC3	glypican 3	ASS1	argininosuccinate synthase 1
FN1	fibronectin 1	STRIP2	striatin interacting protein 2
APOA2	apolipoprotein A2	SEMA4D	semaphorin 4D
ANGPTL3	angiopoietin like 3	CASP14	caspase 14
AHSG	alpha 2-HS glycoprotein	COL7A1	collagen type VII alpha 1 chain
NID1	nidogen 1	ABL1	ABL proto-oncogene 1, non-receptor tyrosine kinase
PROX1	prospero homeobox 1	HEY1	hes related family bHLH transcription factor with YRPW motif 1
SERPINF1	serpin family F member 1	UNC13D	unc-13 homolog D
NR1H4	nuclear receptor subfamily 1 group H member 4	FAS	Fas cell surface death receptor
SDC2	syndecan 2	SEMA3F	semaphorin 3F
ANPEP	alanyl aminopeptidase, membrane	ANXA2P2	annexin A2 pseudogene 2
KNG1	kininogen 1	PTGS1	prostaglandin-endoperoxide synthase 1
SERPINC1	serpin family C member 1	GAL	galanin and GMAP prepropeptide
BAMBI	BMP and activin membrane bound inhibitor	SIRT1	sirtuin 1
SEMA4G	semaphorin 4G	SLC25A6	solute carrier family 25 member 6
SERPINA1	serpin family A member 1	TNFAIP3	TNF alpha induced protein 3
PROC	protein C, inactivator of coagulation factors Va and VIIIa	E2F7	E2F transcription factor 7
COL5A2	collagen type V alpha 2 chain	PALLD	palladin, cytoskeletal associated protein
CDH2	cadherin 2	TLR6	toll like receptor 6
SOX9	SRY-box 9	SEMA3B	semaphorin 3B
FRAS1	Fraser extracellular matrix complex subunit 1	CD97	adhesion G protein-coupled receptor E5
MBL2	mannose binding lectin 2	LAMA3	laminin subunit alpha 3
EPHX2	epoxide hydrolase 2	SYNE2	spectrin repeat containing nuclear envelope protein 2
XBP1	X-box binding protein 1	TNIP1	TNFAIP3 interacting protein 1
CSTA	cystatin A	B4GALT1	beta-1,4-galactosyltransferase 1
GPC6	glypican 6	TLR3	toll like receptor 3
NFATC2	nuclear factor of activated T-cells 2	RPS6KA5	ribosomal protein S6 kinase A5
CD24	CD24 molecule	SEMA3A	semaphorin 3A
ATRN	attractin	SMOC1	SPARC related modular calcium binding 1
CSF3R	colony stimulating factor 3 receptor	GPC5	glypican 5
ANG	angiogenin	CRTAP	cartilage associated protein
PLXNA2	plexin A2	FLNA	filamin A
FLRT2	fibronectin leucine rich transmembrane protein 2	ADAMTS1	ADAM metallopeptidase with thrombospondin type 1 motif 1
SIRPA	signal regulatory protein alpha	IL1RL1	interleukin 1 receptor like 1
NLRP1	NLR family pyrin domain containing 1	RASIP1	Ras interacting protein 1
NRP1	neuropilin 1	FOXC1	forkhead box C1
COL2A1	collagen type II alpha 1 chain	CD44	CD44 molecule (Indian blood group)
IL17RE	interleukin 17 receptor E	ITGA2	integrin subunit alpha 2
TNS3	tensin 3	COL4A5	collagen type IV alpha 5 chain
APOE	apolipoprotein E	PFKP	phosphofructokinase, platelet
LAMB1	laminin subunit beta 1	NMI	N-myc and STAT interactor
FZD5	frizzled class receptor 5	LAMB3	laminin subunit beta 3
TIMP3	TIMP metallopeptidase inhibitor 3	COL12A1	collagen type XII alpha 1 chain
NRP2	neuropilin 2	BMP6	bone morphogenetic protein 6
F11R	F11 receptor	ITGA3	integrin subunit alpha 3
TNFRSF10B	TNF receptor superfamily member 10b	L1CAM	L1 cell adhesion molecule
SYNE2	spectrin repeat containing nuclear envelope protein 2	IFI16	interferon gamma inducible protein 16
OTULIN	OTU deubiquitinase with linear linkage specificity	DNER	delta/notch like EGF repeat containing
KIAA0319	KIAA0319	KLF5	Kruppel like factor 5
PARP4	poly(ADP-ribose) polymerase family member 4	CXCR4	C-X-C motif chemokine receptor 4
SLC16A3	solute carrier family 16 member 3	MSN	moesin
PKM	pyruvate kinase, muscle	F3	coagulation factor III, tissue factor
PLCD3	phospholipase C delta 3	TIMP1	TIMP metallopeptidase inhibitor 1
WWC1	WW and C2 domain containing 1	CD44	CD44 molecule (Indian blood group)
COL4A3	collagen type IV alpha 3 chain	FOLR1	folate receptor 1
IL10RB	interleukin 10 receptor subunit beta	PTGS2	prostaglandin-endoperoxide synthase 2
TICAM1	toll like receptor adaptor molecule 1	ANXA1	annexin A1
SIX4	SIX homeobox 4	SLPI	secretory leukocyte peptidase inhibitor
RHOB	ras homolog family member B	OLR1	oxidized low density lipoprotein receptor 1

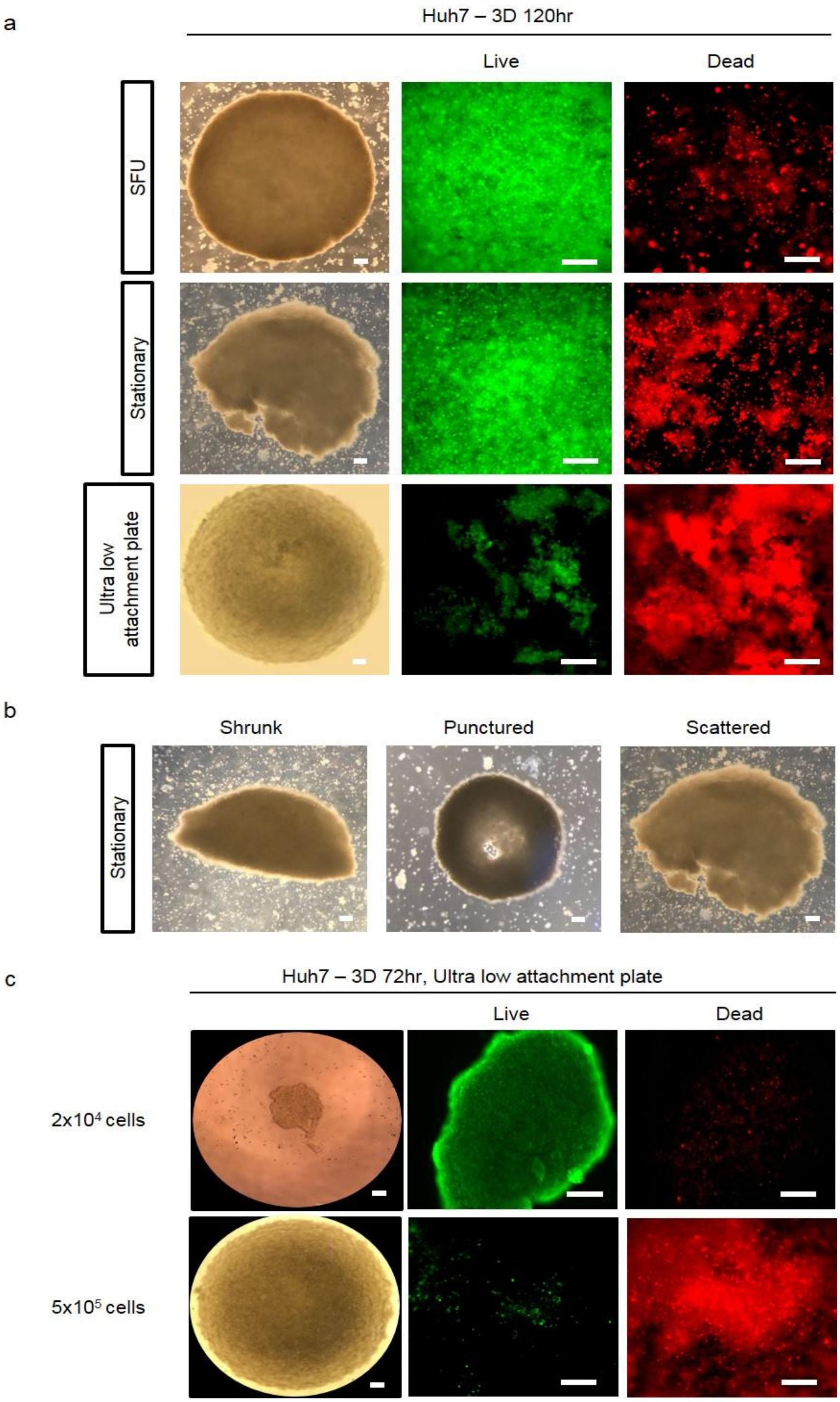
a
Huh7 – 3D 120hr
Live
Dead
SFU
Stationary
Ultra low attachment plate
b
Shrunk
Punctured
Scattered
Stationary
c
Huh7 – 3D 72hr, Ultra low attachment plate
Live
Dead
2x10⁴ cells
5x10⁵ cells

Supp. Fig. S1.

Supplementary figure 1. Comparison between spheroids generated by SFU and other methods.

(a) Representative DIC and Live/Dead images of spheroids generated by SFU, stationary culture, and Ultra-Low Attachment plate at 120 h. Scale bars, 200μm. (b) Appearance of abnormal spheroids in stationary culture at 120 h. Scale bars, 200μm. (c) Representative DIC and Live/Dead images of the spheroid cultured with indicated amounts of cells generated by Ultra-Low Attachment plate for 72 h. Scale bars, 200μm.

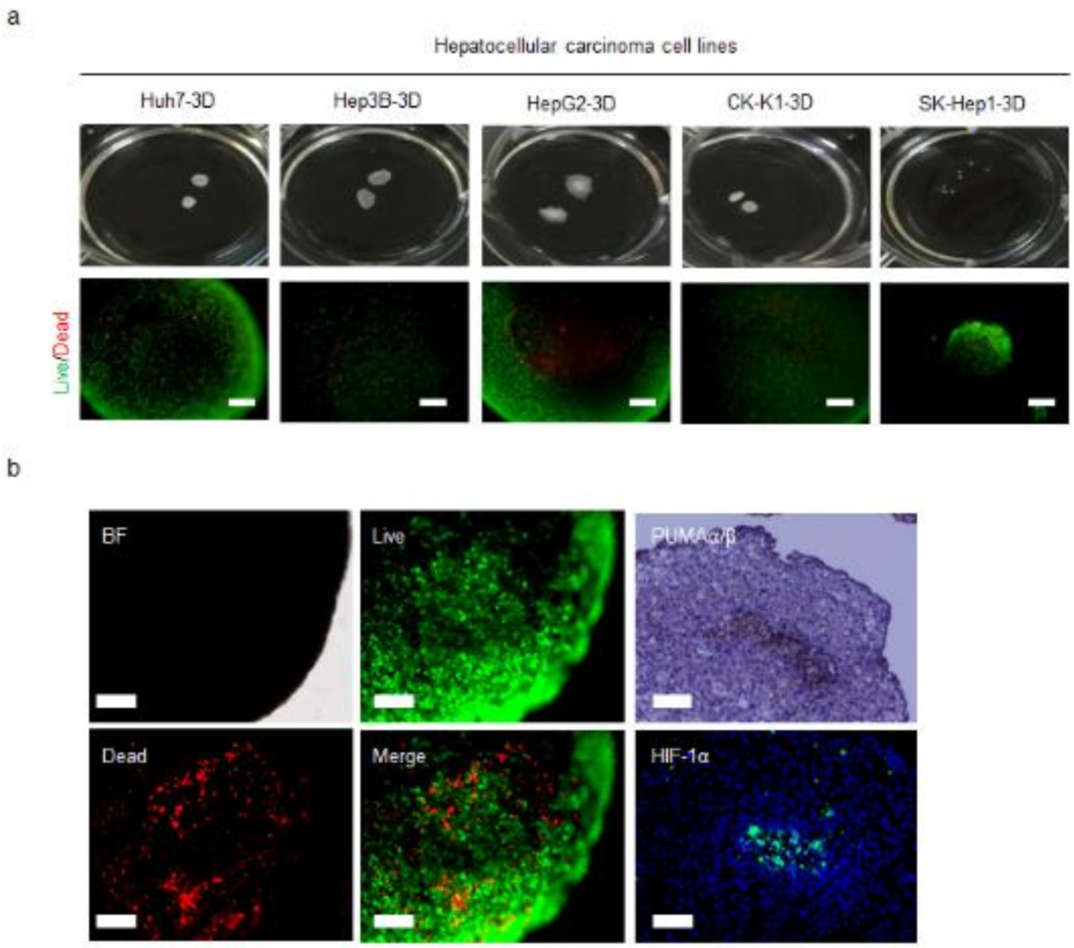

Supplementary figure 2. Various spheroids and its cellular condition in SFU.

(a) Top pictures show the various cell spheroids made by SFU and bottom pictures show fluorescence microscopic image of spheroids after live/dead staining at 120 h. Green and red colours represent living and dead cells, respectively. Scale bars, 200 μm. (b) Representative live/dead staining and immunostaining images of spheroids for PUMAα/β and HIF-1α at 120 h. Scale bars, 200 μm.

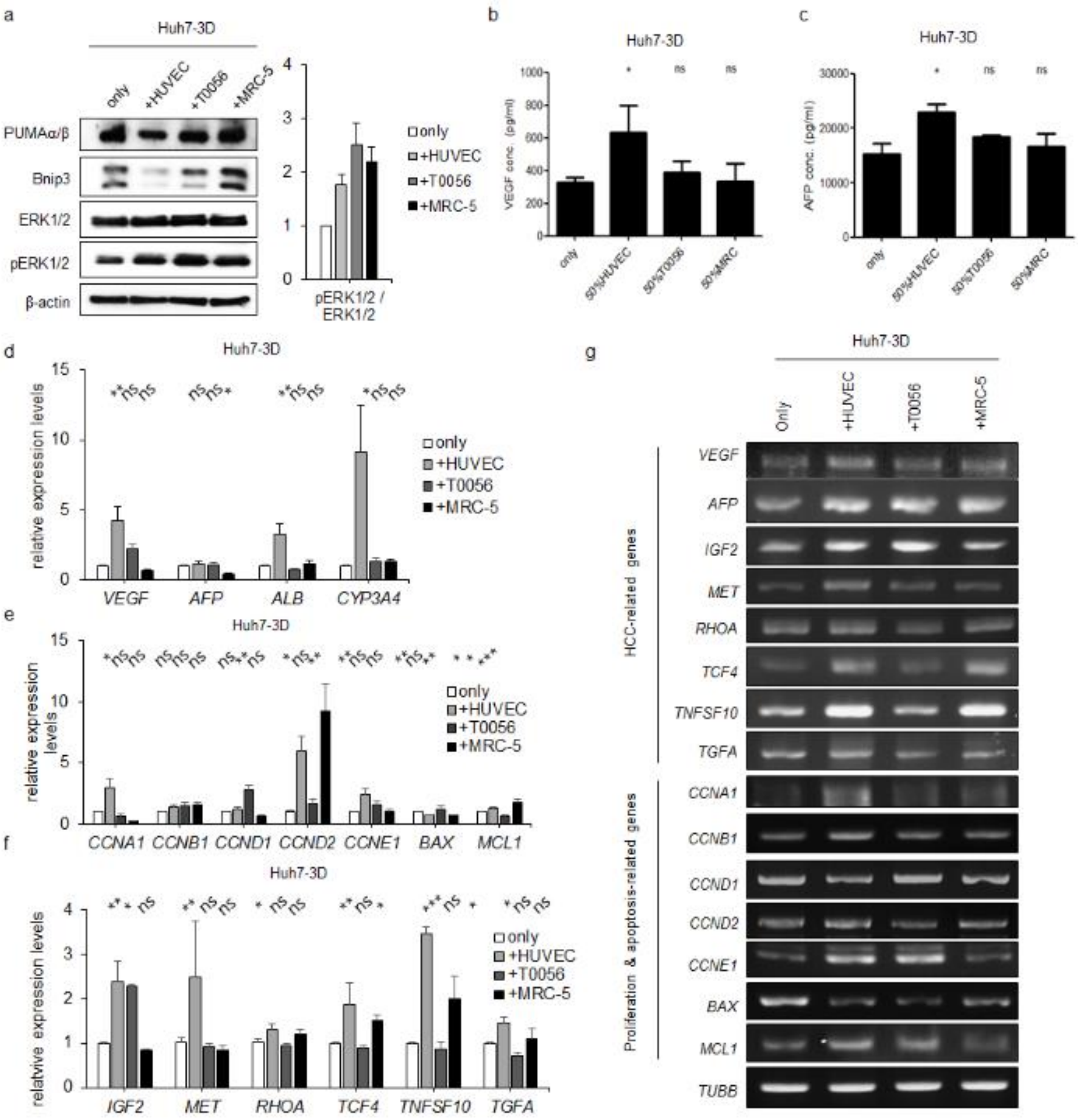

a
Huh7-3D
only
+HUVEC
+T0056
+MRC-5
PUMAα/β
Bnip3
ERK1/2
pERK1/2
β-actin
pERK1/2 / ERK1/2
b
Huh7-3D
VEGF conc. (pg/ml)
c
Huh7-3D
AFP conc. (pg/ml)
d
Huh7-3D
relative expression levels
VEGF
AFP
ALB
CYP3A4
e
Huh7-3D
CCNA1
CCNB1
CCND1
CCND2
CCNE1
BAX
MCL1
f
Huh7-3D
relatvive expression levels
IGF2
MET
RHOA
TCF4
TNFSF10
TGFA
g
Huh7-3D
Only
HCC-related genes
Proliferation & apoptosis-related genes
TUBB

Supp. Fig. S3.

Supplementary figure 3. HUVECs were most effective to improve survival and oncogenic properties of Huh7 spheroids.

(a) Western blot analysis of proteins related to apoptosis and proliferation signalling in spheroids generated by co-culture with HUVECs, T0056, or MRC-5 as compared to Huh7 spheroids generated by monoculture, at 120h (top) and quantitative results (bottom). (b, c) ELISA of VEGF (b) and AFP (c) secretion into culture supernatants by spheroids generated in co-culture with HUVECs, T0056, or MRC-5 as compared with Huh7 spheroids generated by monoculture, at 120h. (d-f) RT-qPCR mRNA expression analysis of *VEGF*, *AFP*, *ALB*, and *CYP3A4* (d), proliferation- (cyclins) and apoptosis (*BAX* and *MCL1*)-related genes (e), and HCC-related genes (*IGF2*, *MET*, *RhoA*, *TCF4*, *TNFSF10*, and TGFα) (f) at day 5. (g) Conventional RT-PCR analysis of *VEGF*, *AFP*, *ALB*, *CYP3A4*, HCC-related genes (*IGF2*, *MET*, *RhoA*, *TCF4*, *TNFSF10*, and TGFα), proliferation- (cyclins) and apoptosis (*BAX* and *MCL1*)-related genes. *P<0.05; **P<0.01; ***P<0.001.

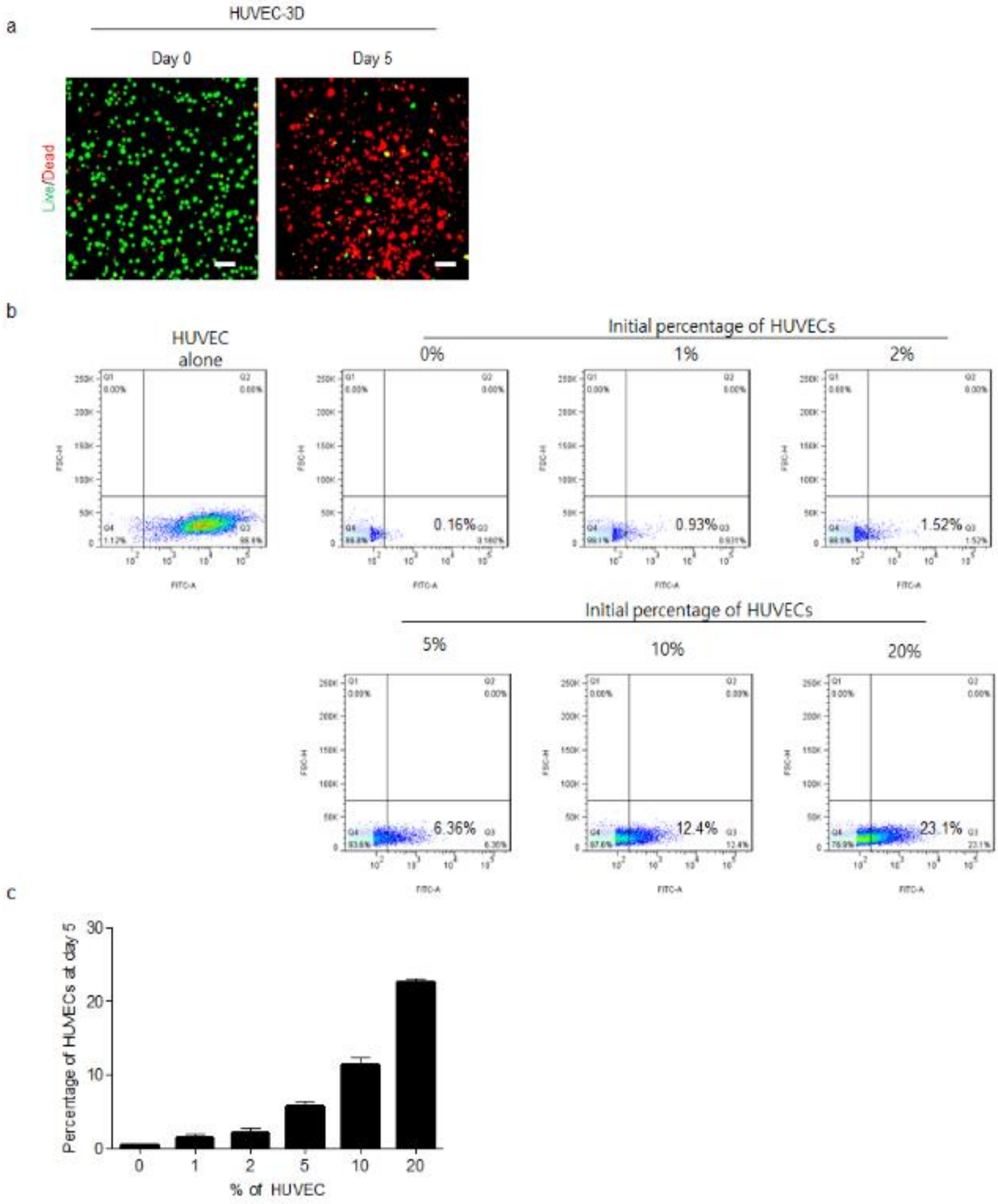
a
HUVEC-3D
Day 0
Day 5
Live/Dead
b
HUVEC alone
Initial percentage of HUVECs
0%
1%
2%
0.16%
0.93%
1.52%
Initial percentage of HUVECs
5%
10%
20%
6.36%
12.4%
23.1%
FITC-A
FSC-H
c
Percentage of HUMECs at day 5
% of HUVEC

Supp. Fig. S4.

Supplementary figure 4. HUVECs maintained the initial proportion in Huh7 spheroids for 5 days.

(a) Fluorescence microscopic image of HUVECs spheroids after live/dead staining at day 0 and 5. Green and red colours represent living and dead cells, respectively. Scale bar, 200μm. (b) Flow cytometry analysis for showing the proportion of GFP-tagged HUVECs in the spheroids at day 5. (c) Percentage of GFP expression cells in the Huh7 spheroids co-cultured with indicated percentage of HUVECs.

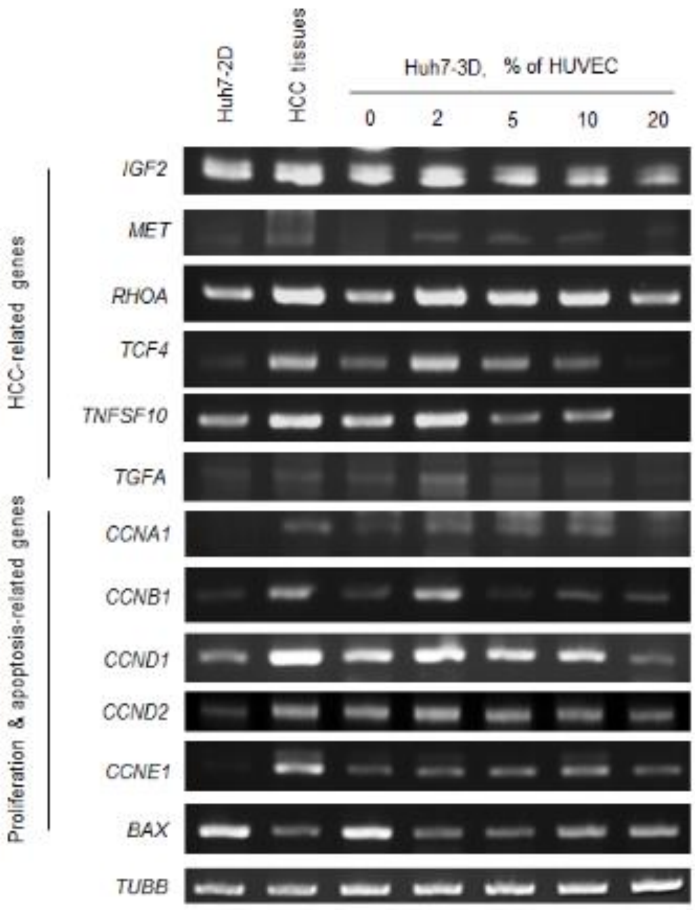

Supplementary figure 5. RT-PCR analysis generated by Huh7 spheroids co-cultured with various percentage of HUVECs.

Conventional RT-PCR analysis of HCC-related genes (*IGF2*, *MET*, *RhoA*, *TCF4*, *TNFSF10*, and TGFα), proliferation- (cyclins) and apoptosis (*BAX* and *MCL1*)-related genes generated by Huh7 spheroids co-cultured with indicated percentage of HUVECs as compared to Huh7 monolayers and human liver cancer tissue.

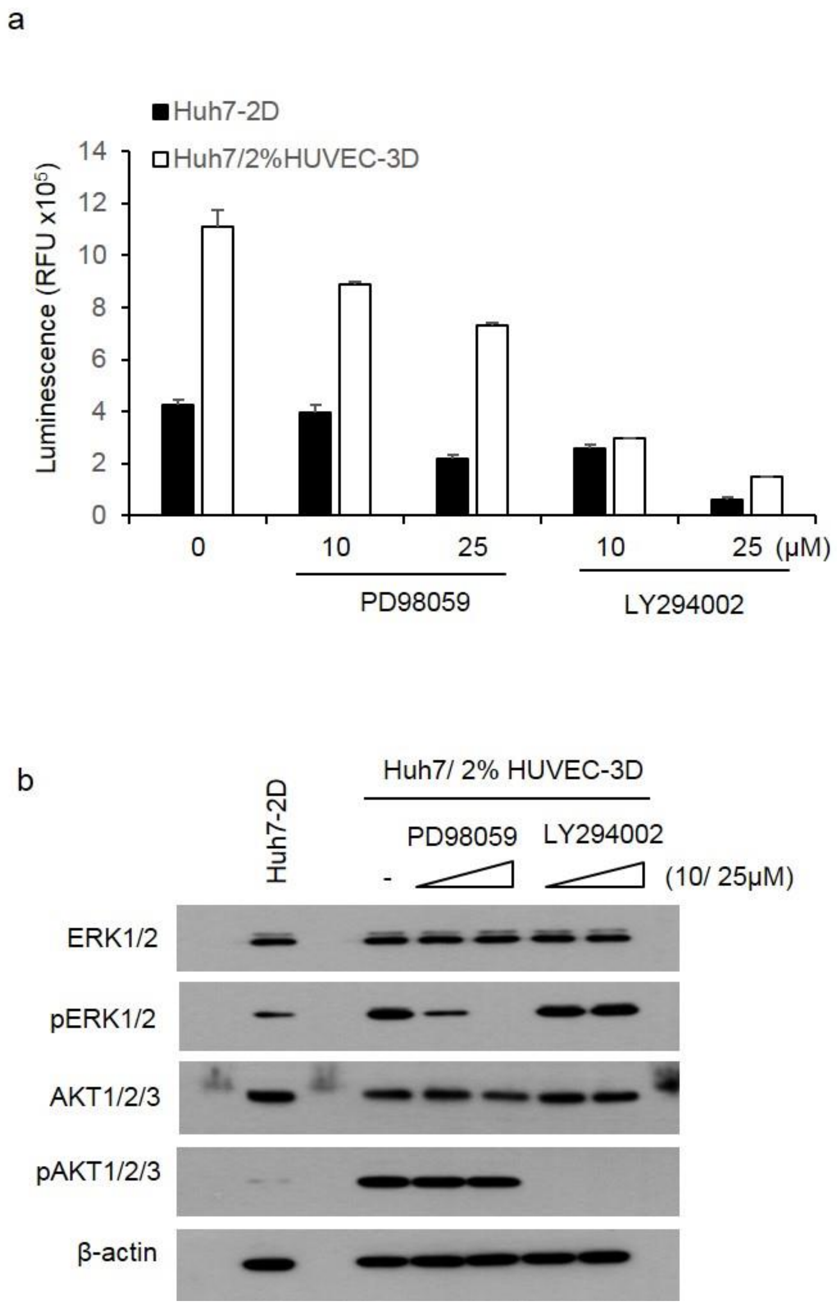
a
Huh7-2D
Huh7/2%HUVEC-3D
Luminescence (RFU x10⁵)
14
12
10
8
6
4
2
0
0
10
25
10
25 (μM)
PD98059
LY294002
b
Huh7-2D
Huh7/ 2% HUVEC-3D
PD98059
LY294002
-
(10/ 25μM)
ERK1/2
pERK1/2
AKT1/2/3
pAKT1/2/3
β-actin

Supp. Fig. S6.

Supplementary figure 6. Huh7 spheroids co-cultured with HUVECs increased anti-apoptotic effect via the activation of MAPK and PI3K-Akt signalling pathway.

(a) Huh7 cells (Huh7-2D) were seeded into the wells of the plate at 3 x10^3 cells and incubated for 4 days and Huh7/2% HUVEC-3D spheroids were transferred a single spheroid per well in 96 well round-bottomed plate. Huh7-2D and Huh7/2% HUVEC-3D spheroids were treated with either DMSO, PD98059, or LY294002 at indicated concentration for 72 h. The viability of Huh7-2D and Huh7/2% HUVEC-3D spheroids were measured by the cellTiter-Glo assay. (b) At 72 h after inhibitors treatment, Huh7-2D (untreated) and Huh7/2% HUVEC-3D spheroids of each experimental group were analyzed by western blotting with indicated antibodies.

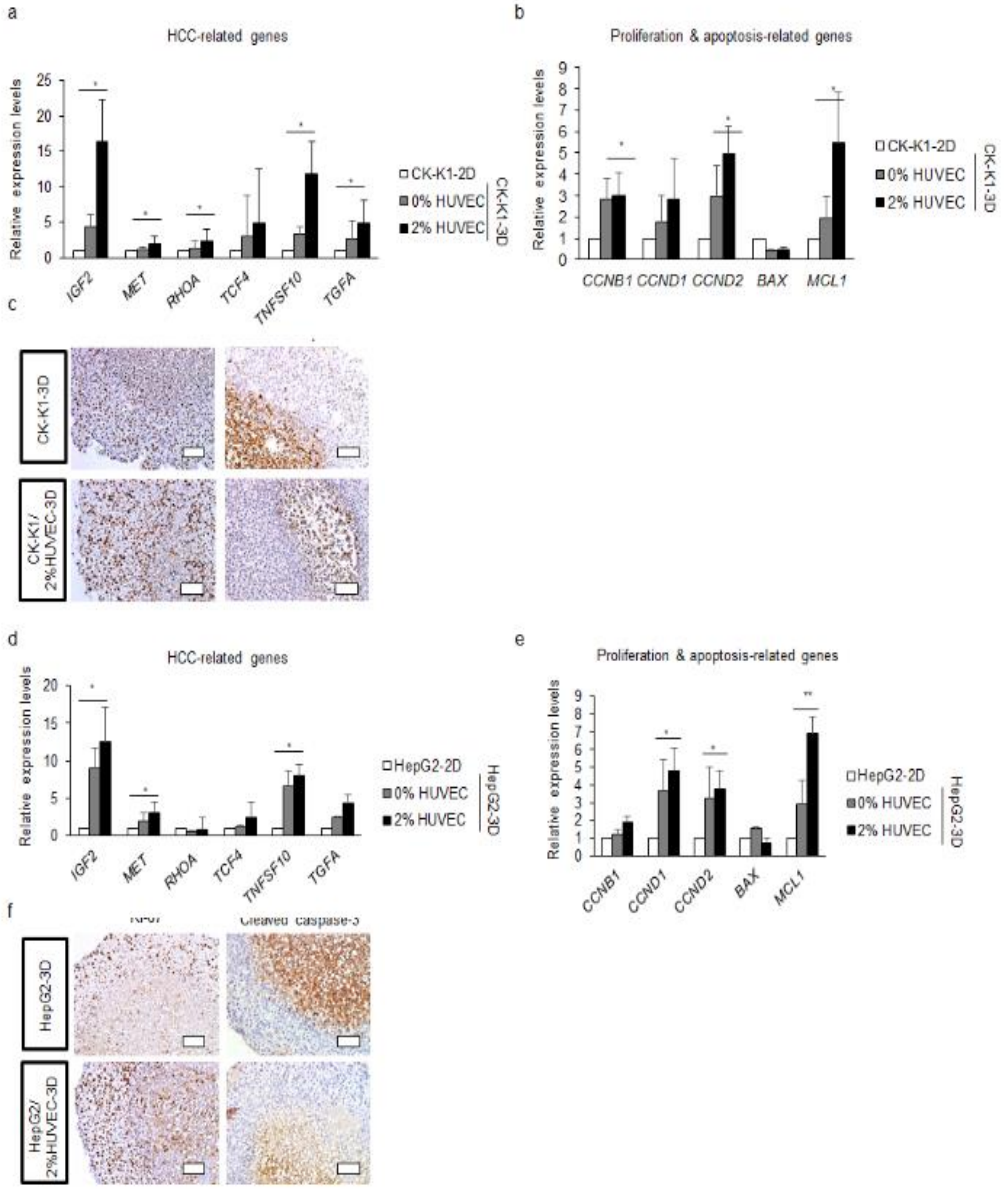
a
HCC-related genes
Relative expression levels
IGF2
MET
RHOA
TCF4
TNFSF10
TGFA
CK-K1-2D
0% HUVEC
2% HUVEC
CK-K1-3D
b
Proliferation & apoptosis-related genes
CCNB1
CCND1
CCND2
BAX
MCL1
c
CK-K1-3D
CK-K1/ 2%HUVEC-3D
d
HepG2-2D
HepG2-3D
e
f
HepG2/ 2%HUVEC-3D

Supp. Fig. S7.

Supplementary figure 7. HUVECs enhance the survival of spheroids of other liver cancer cell lines

(a, d) RT-qPCR analysis of tumour-related genes in the CKK-1 (a) and HepG2 (d) spheroids co-cultured with the indicated percentages of HUVECs at 120 h in comparison to cancer/normal liver tissues and monolayers (2D). (b, e) Proliferation (cyclins and AKT/ERK pathway) and apoptosis signalling (*BAX* and *MCL1*)-related mRNA as detected by RT-qPCR. (c, f) IHC images of CKK-1-3D or CKK-1 / 2% HUVEC-3D (c) and HepG2-3D or HepG2 / 2% HUVEC-3D for proliferation (Ki-67) and apoptosis (cleaved caspase-3) markers. Scale bars, 200 μm.

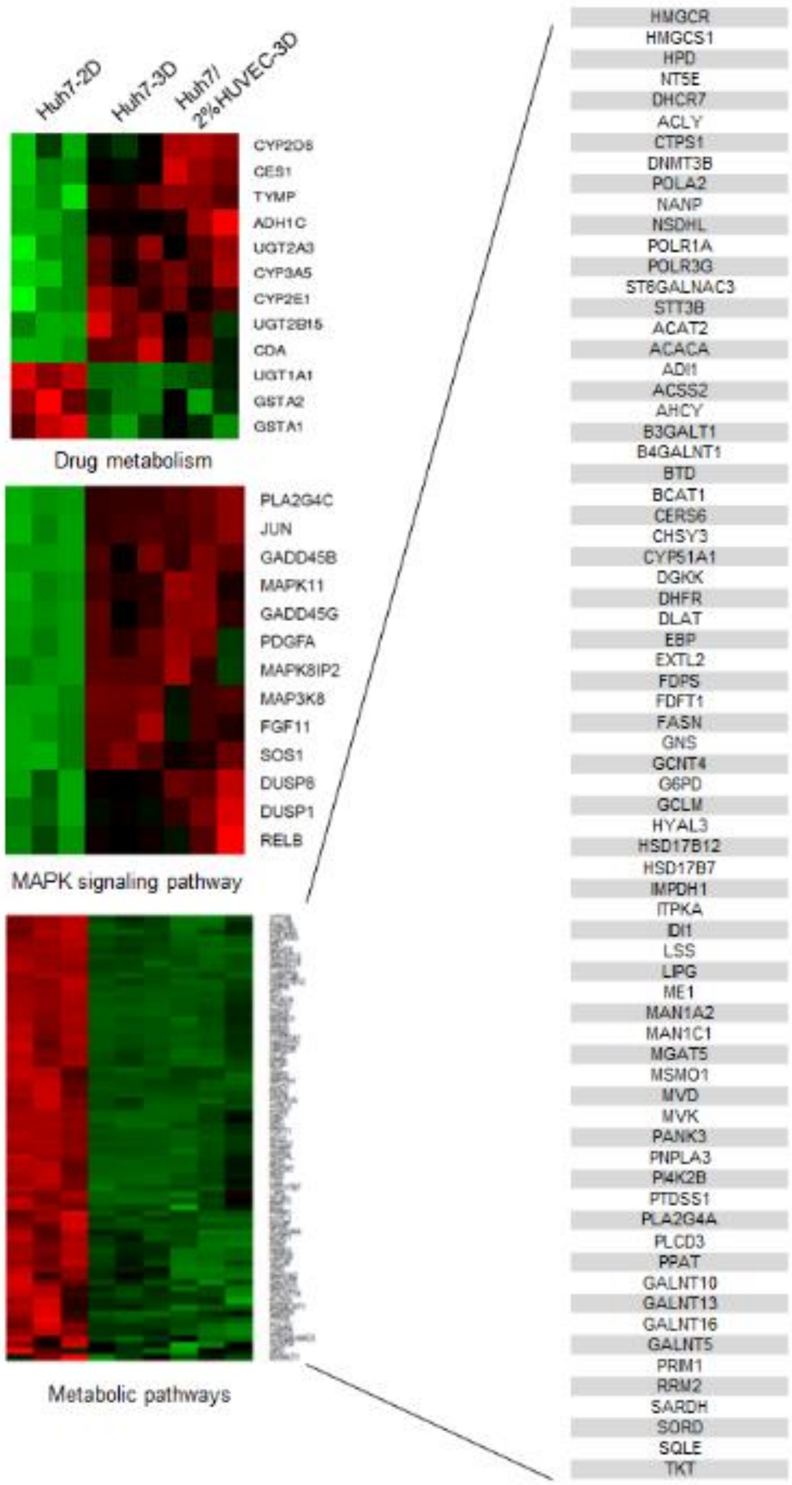
Huh7-2D
Huh7-3D
Huh7/ 2%HUVEC-3D
CYP2D6
CES1
TYMP
ADH1C
UGT2A3
CYP3A5
CYP2E1
UGT2B15
CDA
UGT1A1
GSTA2
GSTA1
Drug metabolism
PLA2G4C
JUN
GADD45B
MAPK11
GADD45G
PDGFA
MAPK8IP2
MAP3K8
FGF11
SOS1
DUSP6
DUSP1
RELB
MAPK signaling pathway
Metabolic pathways
HMGCR
HMGCS1
HPD
NT5E
DHCR7
ACLY
CTPS1
DNMT3B
POLA2
NANP
NSDHL
POLR1A
POLR3G
ST6GALNAC3
STT3B
ACAT2
ACACA
ADI1
ACSS2
AHCY
B3GALT1
B4GALNT1
BTD
BCAT1
CERS6
CHSY3
CYP51A1
DGKK
DHFR
DLAT
EBP
EXTL2
FDPS
FDFT1
FASN
GNS
GCNT4
G6PD
GCLM
HYAL3
HSD17B12
HSD17B7
IMPDH1
ITPKA
IDI1
LSS
LIPG
ME1
MAN1A2
MAN1C1
MGAT5
MSMO1
MVD
MVK
PANK3
PNPLA3
PI4K2B
PTDSS1
PLA2G4A
PLCD3
PPAT
GALNT10
GALNT13
GALNT16
GALNT5
PRIM1
RRM2
SARDH
SORD
SQLE
TKT

Supp. Fig. S8.

Supplementary figure 8. Representative heatmap of the comparative expression level of mRNA in Huh7-2D, Huh7-3D and Huh7/2% HUVEC-3D spheroids. Drug metabolism in a group I, MAPK signalling pathway in a group II, up regulated genes in Huh7/2% HUVEC-3D spheroids, and metabolism pathway in a group III down regulated genes in Huh7/2% HUVEC-3D spheroids.

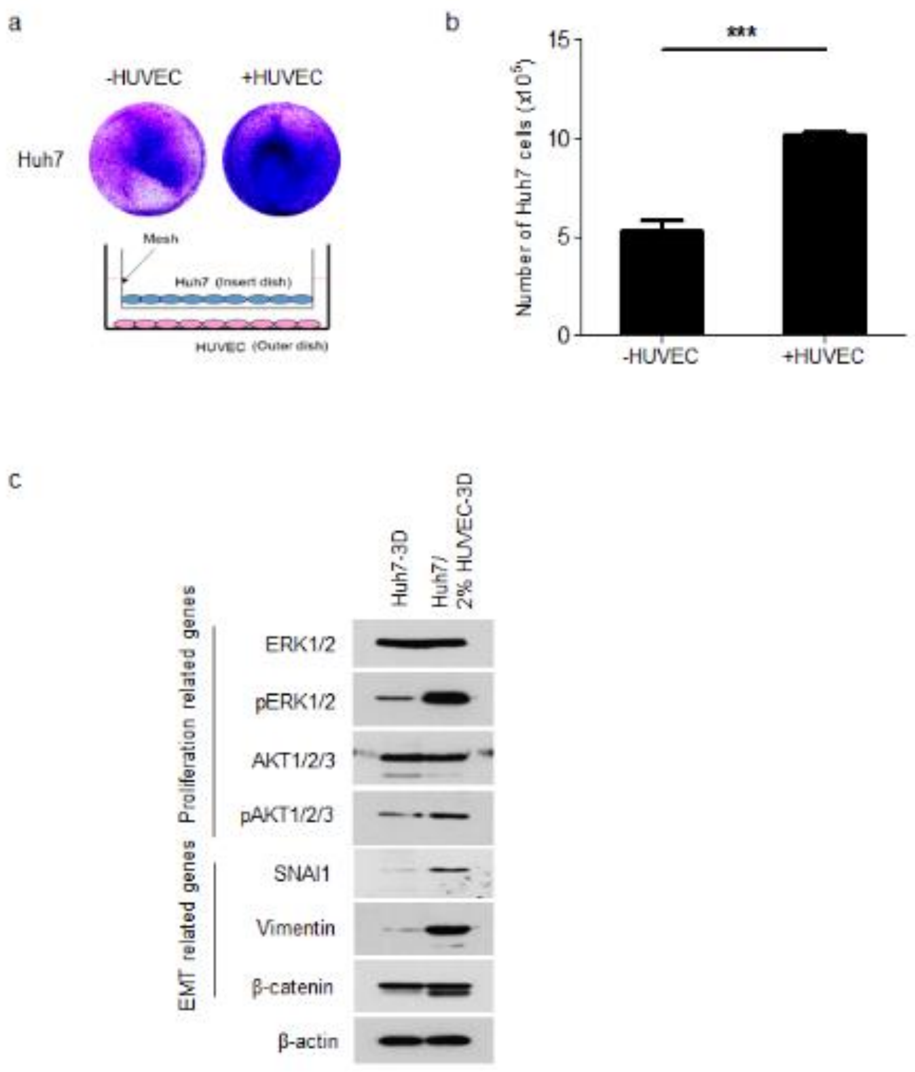

Supplementary figure 9. Proliferation assay using 2D permeable dish and 3D Huh7 spheroids.

(a) Crystal violet staining of Huh7 cells cultured with/without HUVECs in a bi-culture dish designed in-house (top). Illustration of the co-culture test using the bi-culture plate (bottom). (b) Number of Huh7 cells cultured with/without HUVECs in a bi-culture dish. (c) The spheroids of each group for invasion assay were cultured for 120 h (hanging drop for 48 h and rotatory culture for 72 h) and analysed by western blotting with antibodies of selected proliferation and EMT-related genes.

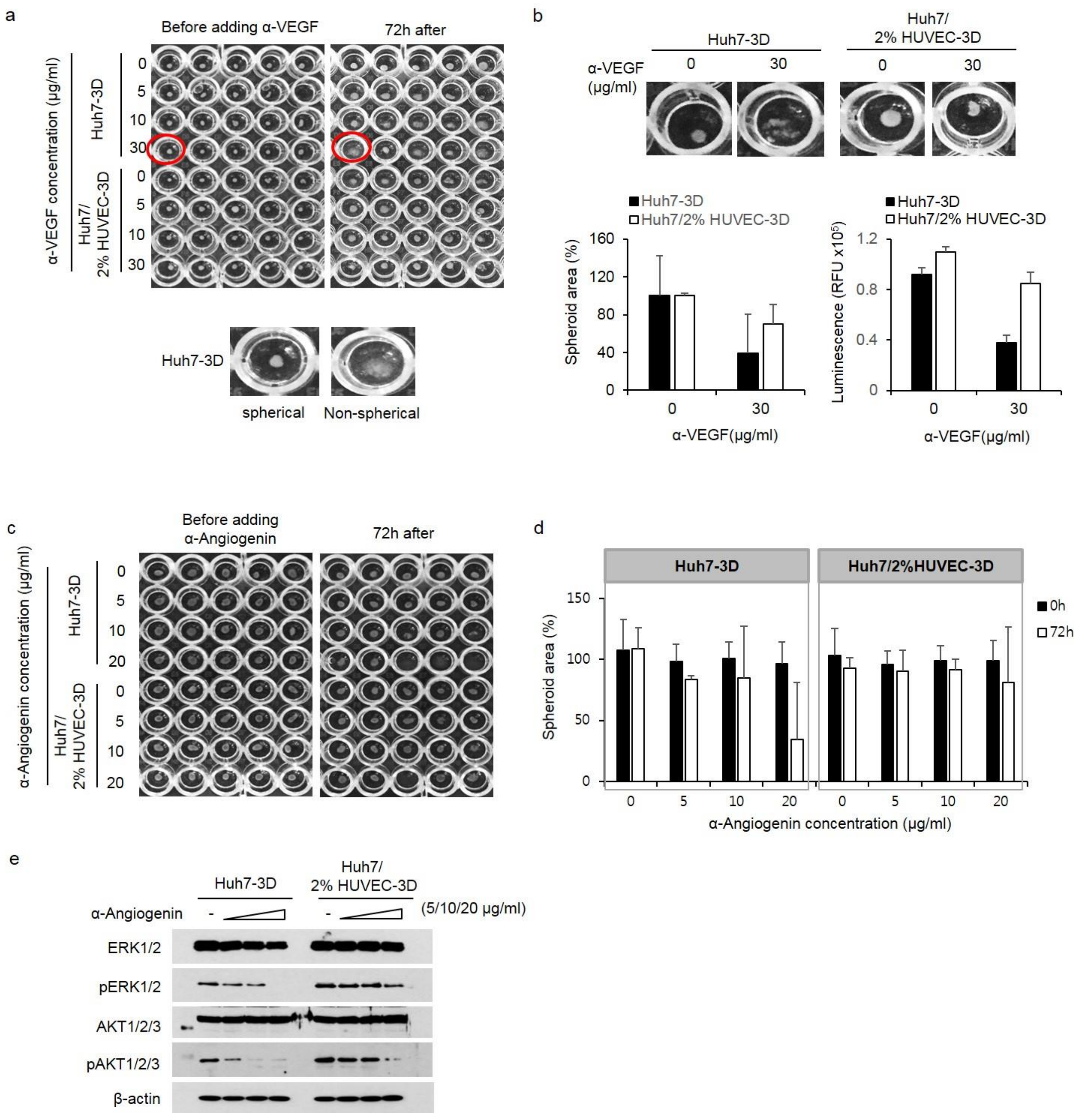
a
Before adding α-VEGF
72h after
α-VEGF concentration (μg/ml)
Huh7-3D
Huh7/ 2% HUVEC-3D
0
5
10
30
Huh7-3D
spherical
Non-spherical
b
Huh7-3D
Huh7/ 2% HUVEC-3D
α-VEGF (μg/ml)
0
30
Huh7-3D
Huh7/2% HUVEC-3D
Spheroid area (%)
Luminescence (RFU x10^5)
α-VEGF(μg/ml)
c
Before adding α-Angiogenin
72h after
α-Angiogenin concentration (μg/ml)
20
d
Huh7-3D
Huh7/2%HUVEC-3D
Spheroid area (%)
0h
72h
α-Angiogenin concentration (μg/ml)
e
α-Angiogenin
(5/10/20 μg/ml)
ERK1/2
pERK1/2
AKT1/2/3
pAKT1/2/3
β-actin

Supp. Fig. S10.

Supplementary figure 10. Blocking of VEGF and angiogenin by specific antibodies inhibited survival of tumouroids.

(a) Huh7-3D, and Huh7/2% HUVEC-3D spheroids were transferred a single spheroid on each well of 96 well plate and were treated with serum-free DMEM medium containing VEGF at indicated concentration for 72 h. Representative image of survival of the spheroid was shown at before adding VEGF antibody (0 h) and 72 h after antibody treatment. (b) The viability of Huh7 spheroids measured by quantification of spheroid area (%) using ImageJ software (left) and the CellTiter-Glo assay (right). (c) Huh7-3D, and Huh7/2% HUVEC-3D spheroids were treated with angiogenin neutralizing antibody at indicated concentration for 72 h. Representative Image of survival in Huh7 spheroids was shown at 0h and 72 h after antibody treatment. (d) Spheroid areas were quantitated using image J software at before adding an angiogenin antibody (0h) and after 72 h. Error bars display the standard deviation of five independent measurements. (e) At 72 h after angiogenin antibody treatment, spheroids of each experimental group were harvested and analyzed by western blotting with indicated antibodies.

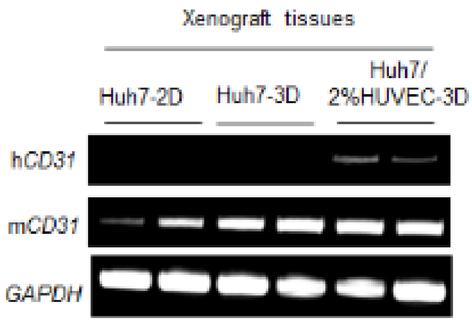

Supplementary figure 11. RT-PCR analysis of CD31 in tissues obtained from nude mice.

The existence of HUVECs were analysed by RT-PCR with a human and mouse CD31-specific primer set in Huh7 monolayers (2D), Huh7 spheroids (-HUVECs), and Huh7 tumouroids with 2% of HUVECs (+HUVECs).

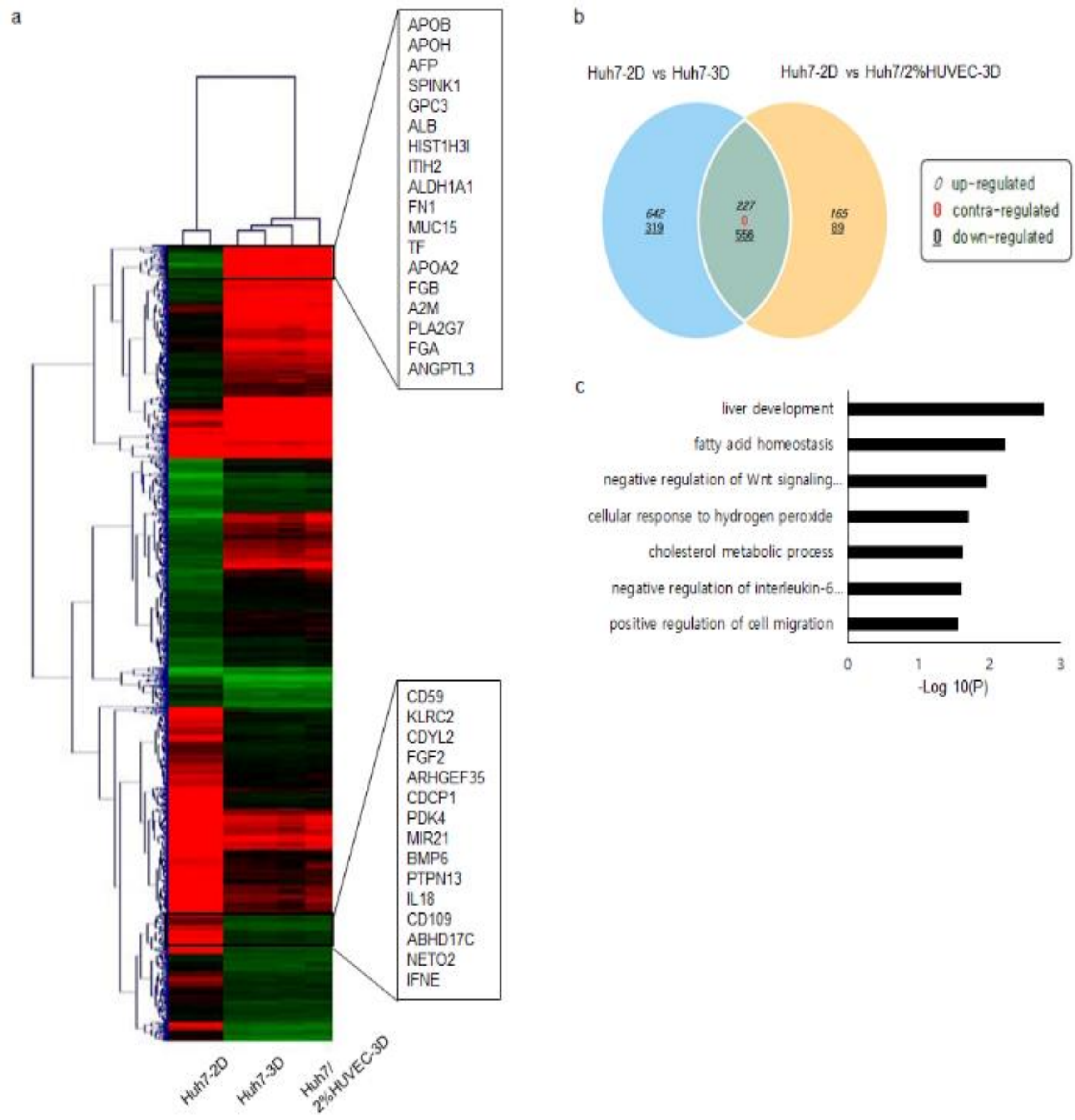

Supplementary figure 12. Microarray analysis of tissues obtained from nude mice.

(a) Heatmap analysis of the comparative expression level of RNA in tumour tissue from nude mice injected Huh7-2D, Huh7-3D, and Huh7/2% HUVEC-3D. Color indicates the degree of up-regulation (red) and down-regulation (green) in RNA levels. (b) Venn diagram of depicting changes in Huh7-2D, Huh7-3D, and Huh7/2% HUVEC-3D. (c) Gene ontology analysis of 254 genes differentially expressed in only Huh7/2% HUVEC-3D. The graph shows the minus log p-values for the enrichment of a specific pathway.

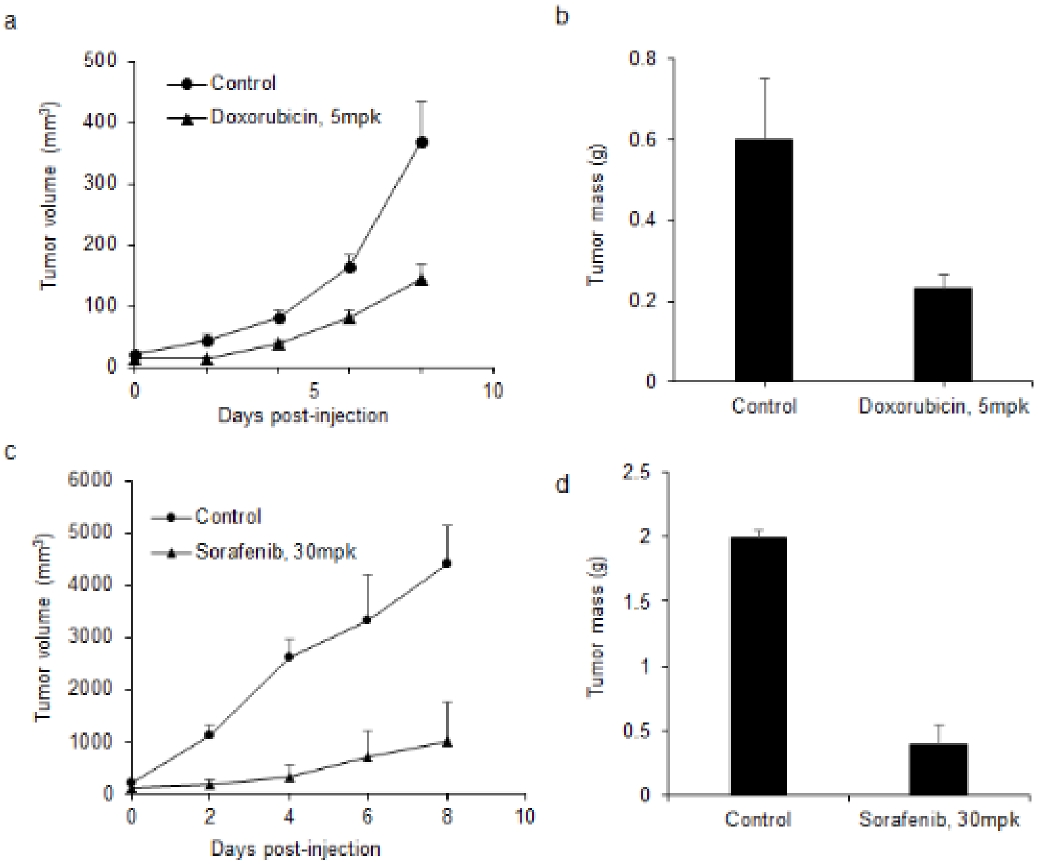

Supplementary figure 13. ***In vivo*** **anti-cancer efficacy test using monolayer Huh7 cells.**

In vivo efficacy test using monolayer Huh7 cells, was tested using Doxorubicin and Sorafenib administered orally at indicated concentration (a, b) Changes in tumour volume and mass after treatment with Doxorubicin (a) and Sorafenib (b) as compared to the control were measured for 8 days.

Fig. 1g

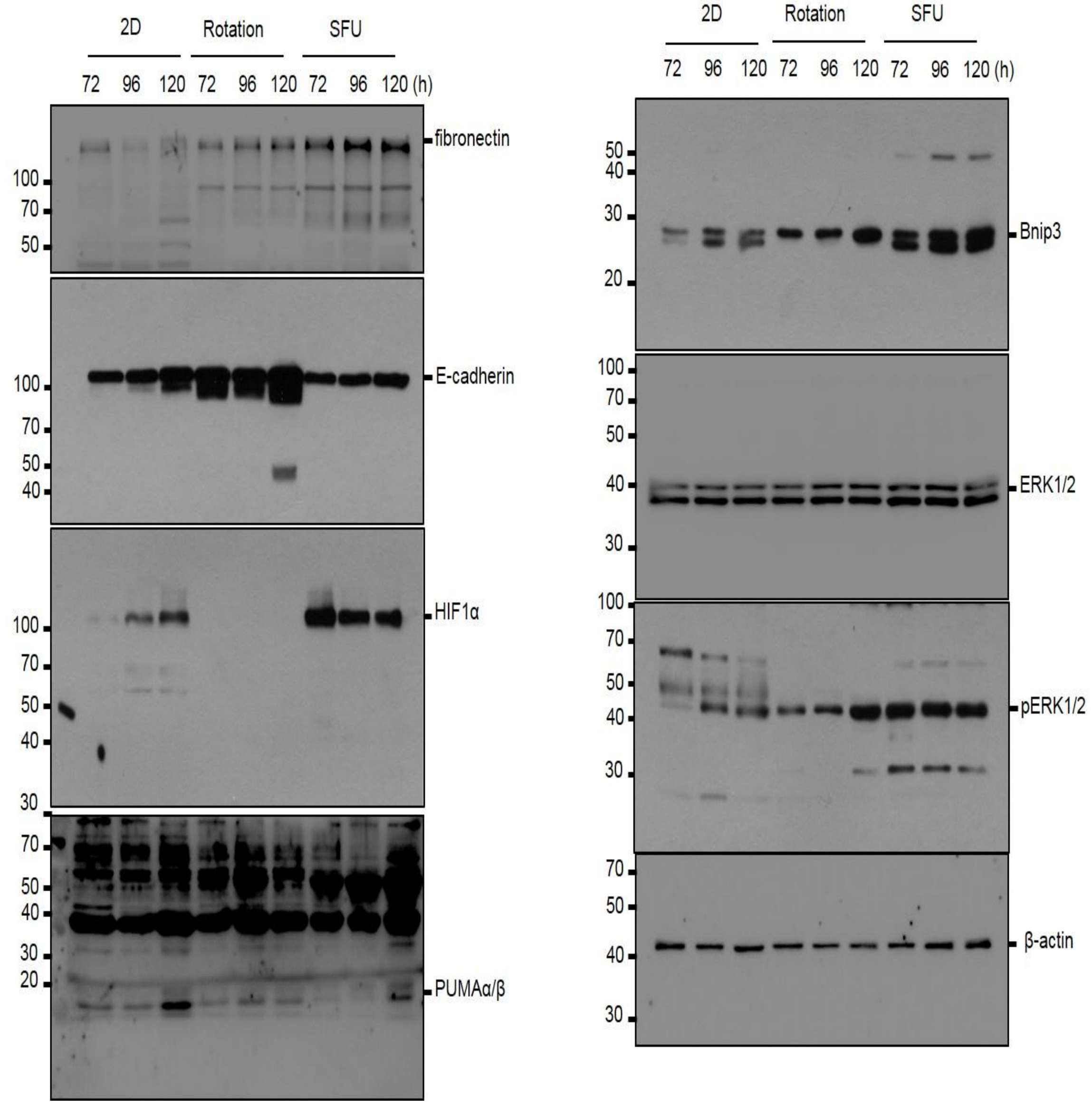

Supplementary figure 14. Uncropped images.

Fig. 2c

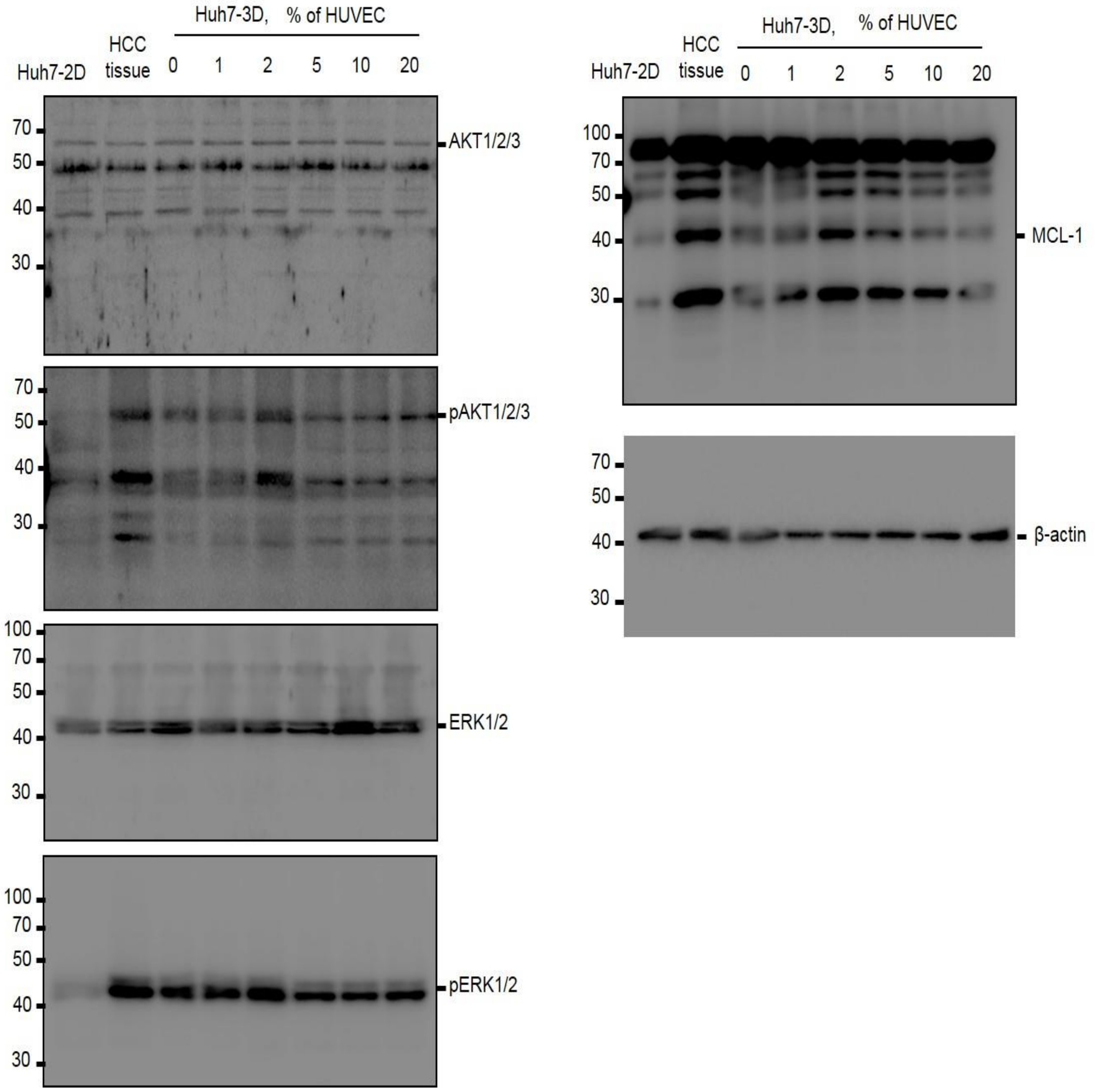

Supplementary figure 14. Uncropped images (Continue).

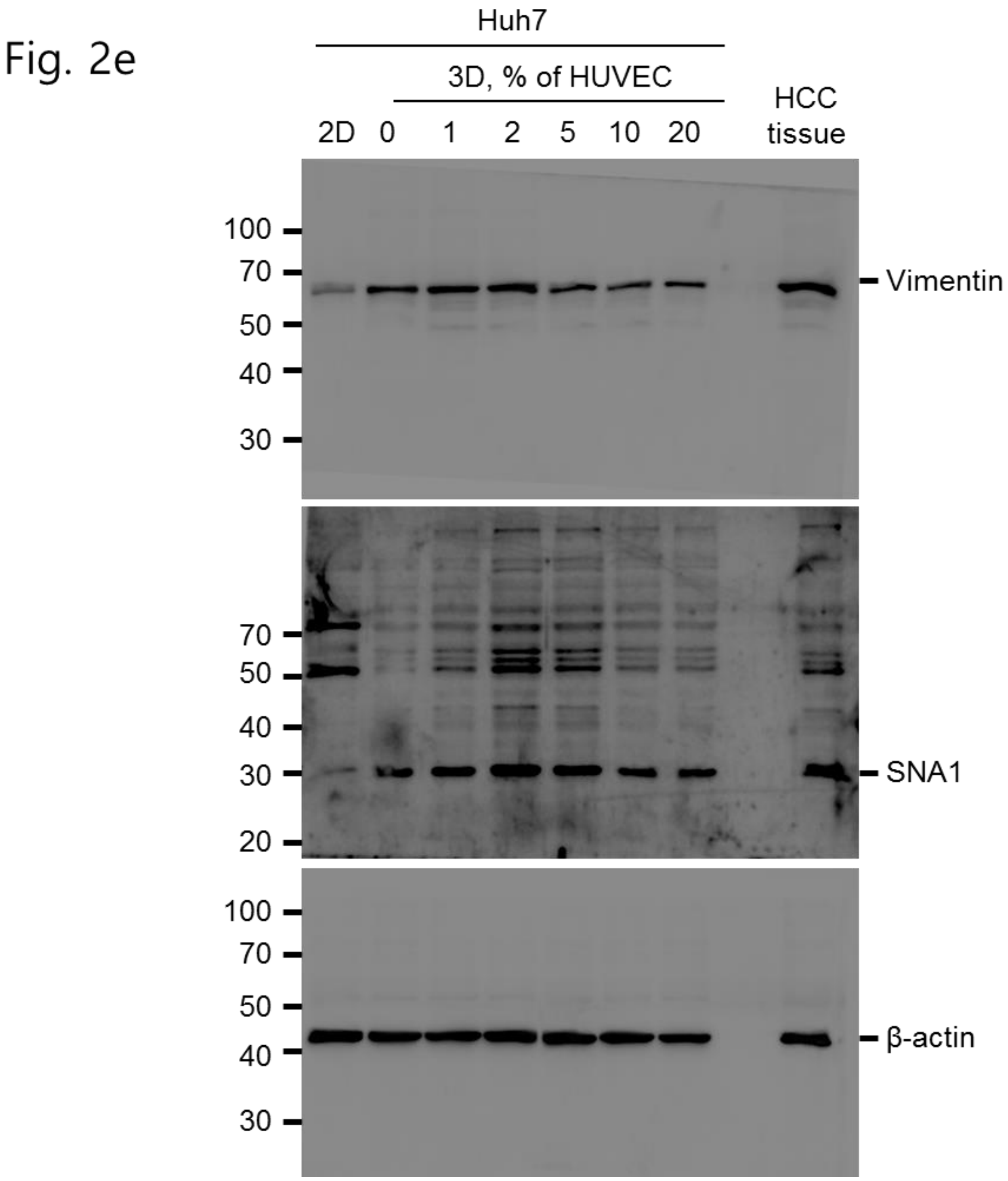

Supplementary figure 14. Uncropped images (Continue).

Fig. 4e

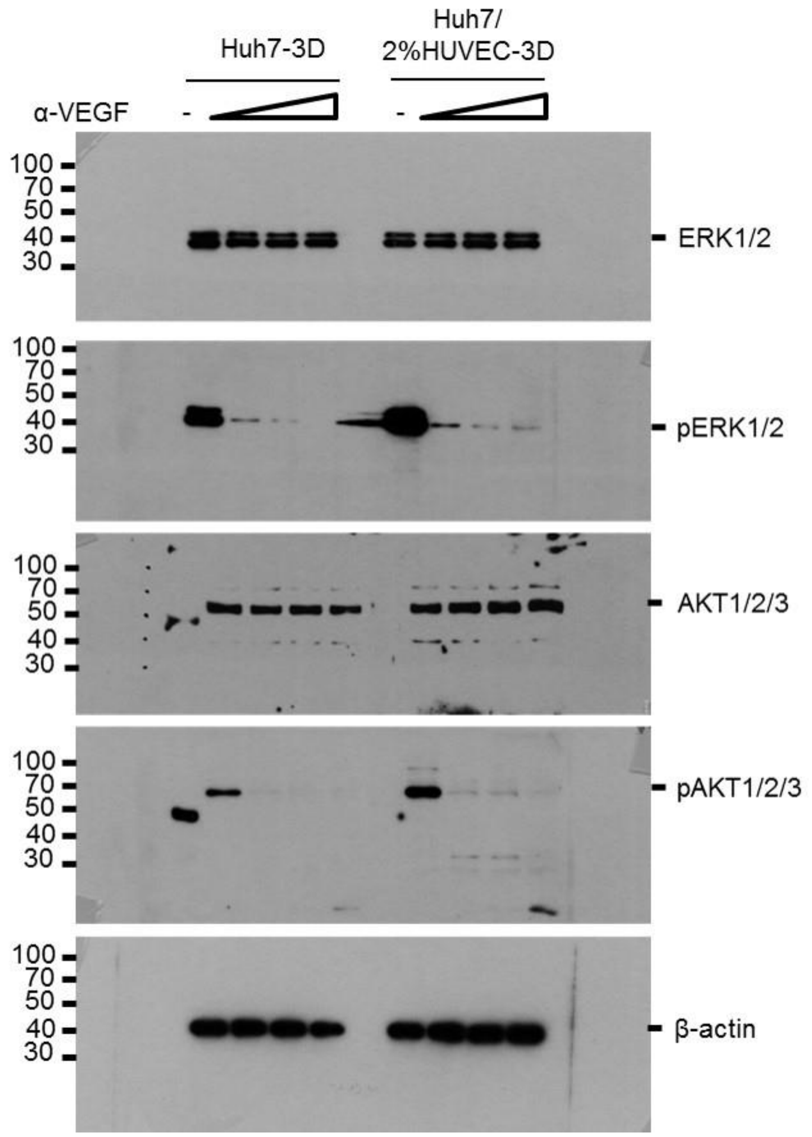

Supplementary figure 14. Uncropped images (Continue).

Fig. 6c

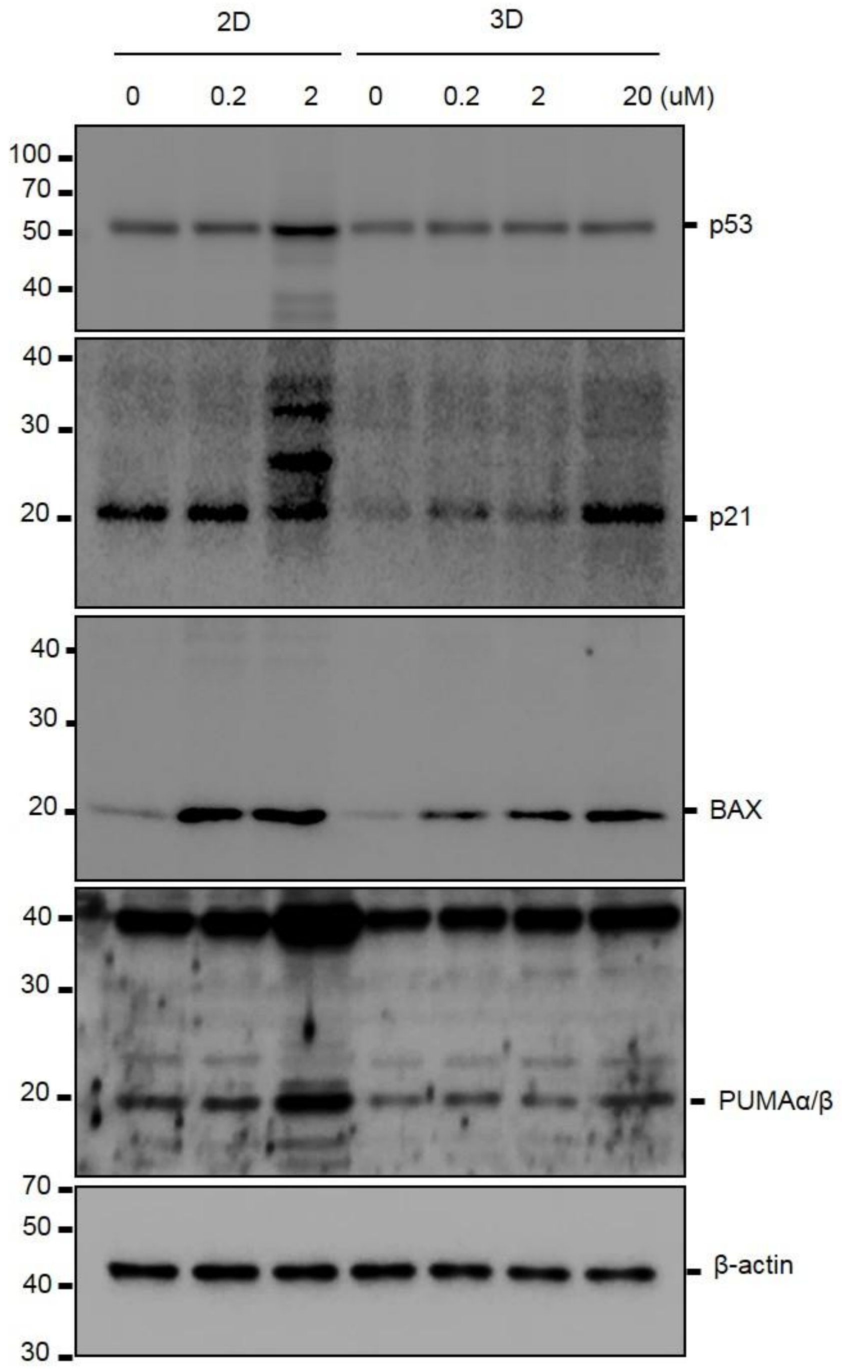

Supplementary figure 14. Uncropped images (Continue).

Fig. 6d

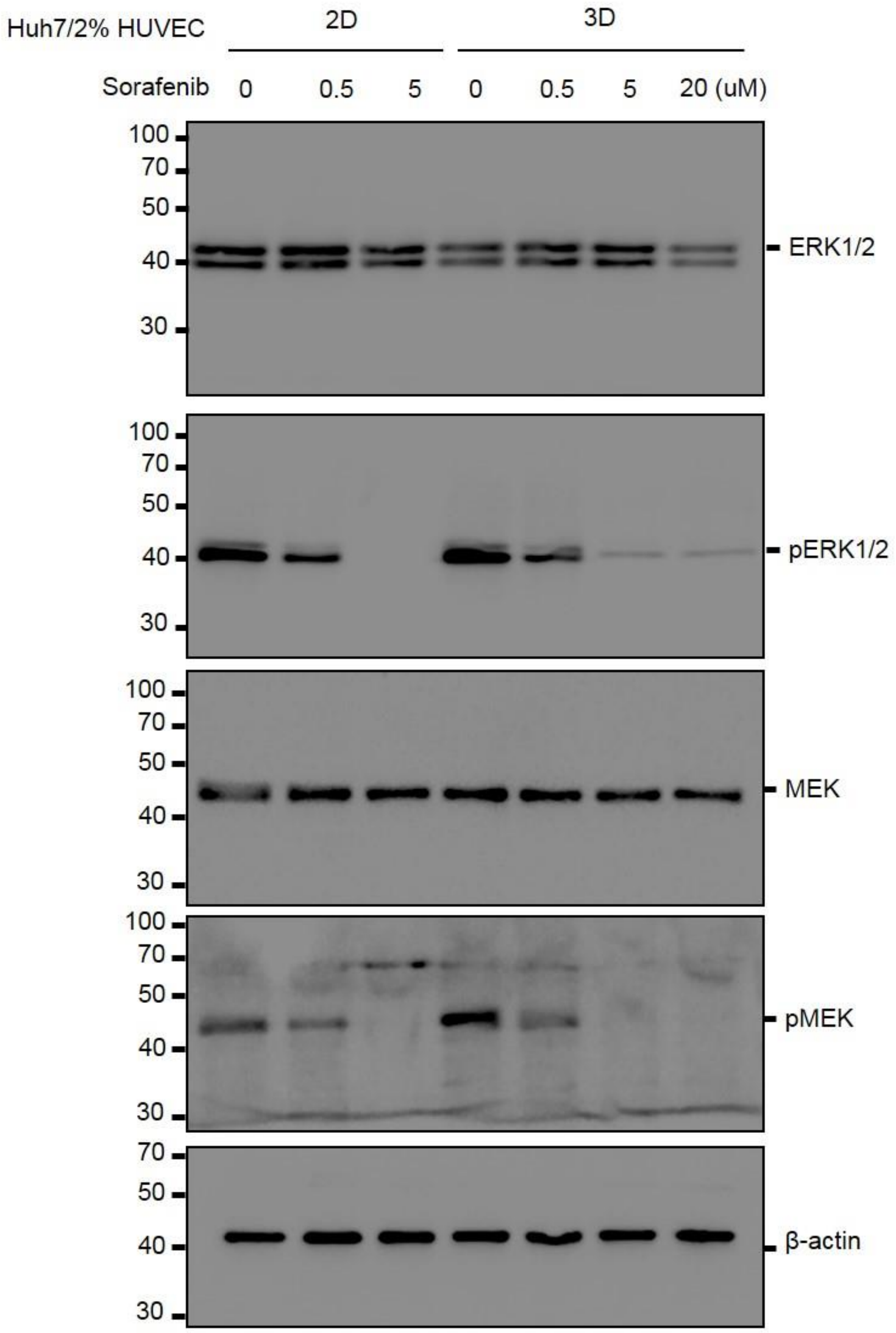

Supplementary figure 14. Uncropped images (Continue).
